# Bioinspired Porphyrin‐Based Catalysts From Temporospatial Confined Frameworks for Ultrafast Removal of Sulfaclozine

**DOI:** 10.1002/advs.77224

**Published:** 2026-08-30

**Authors:** Yuxin Lu, Yu‐Hang Li, Zhiyi Sun, Fanke Tong, Zelin Li, Sheng Wang, Shangkun Pei, Yi Li, Xiyan Xu, Chong‐Chen Wang, Bo Wang, Xiang Li

**Affiliations:** ^1^ School of Chemistry and Chemical Engineering Beijing Institute of Technology Beijing People's Republic of China; ^2^ Eco‐environment and Resource Efficiency Research Laboratory School of Environment and Energy Peking University Shenzhen Graduate School Shenzhen Guangdong People's Republic of China; ^3^ Beijing Key Laboratory of Functional Materials For Building Structure and Environment Remediation Beijing University of Civil Engineering and Architecture Beijing People's Republic of China

**Keywords:** algae‐inspired, confinement, defect engineering, metal‐organic framework, photocatalysis

## Abstract

Inspired by natural photosynthetic systems, the rational design of artificial photocatalysts featuring asymmetrically coordinated dual‐centers and a tailored microenvironment offers promising opportunities to overcome the intrinsic limitations in selective chemical reactions. Here, we present an asymmetric coordination catalyst by immobilizing porphyrins within a defect‐engineered metal–organic framework. By leveraging the matched size of guest molecules in heterogeneous pores, the electron‐hole recombination obstacle was significantly overcome. Across the library of multivariate zirconia metal‐organic frameworks, the optimal member stands out for its photocatalytic properties, delivering an excellent apparent quantum yield (11.31%) for ultra‐fast degradation of sulfaclozine (SCL) with a 10‐minute reaction time. Consistent with the extended X‐ray absorption fine structure (EXAFS) and theoretical calculations, the confined groups in microporous environments remarkably increased the charge separation ability of the Zr‐O nano‐capsules in biomimetic porous PSI and PSII systems. The turnover frequency is 32.1 and 36.9 times higher than that of the pristine framework and the commercial TiO_2_ photocatalyst, respectively. This work establishes a broadly applicable strategy for multivariate zirconia metal‐organic frameworks and provides a platform for tailoring active‐site configuration for photocatalytic reactions.

## Graphical abstract

We report the rational design of artificial photocatalysts with asymmetrically coordinated dual‐metal centers and a tailored microenvironment, broadly applicable strategy is established for the synthesis of multivariate zirconium‐based metal–organic frameworks, offering a platform for precisely tuning active‐site configurations to optimize photocatalytic performance.

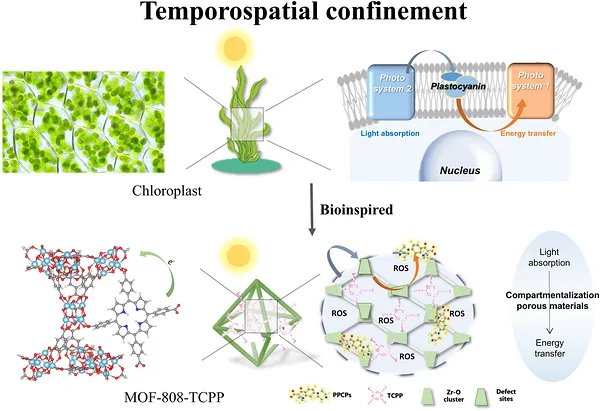

## 1 Introduction

With the growing demand for sustainable development, the pervasive contamination of water resources by organic micropollutants (OMPs) poses an urgent threat to ecosystems and human health [1, 2, 3]. Chloride‐containing antibiotics are a class of anthropogenic chemicals that have been extensively consumed in medical factories, agricultural processes, and chemical industries. Wastewater from these industries contains increasing amounts of micropollutants, which are toxic and not amenable to conventional secondary treatment in sewage treatment plants (STPs), and potentially cause severe impacts on human health and the ecosystem [4, 5]. Heterogeneous photocatalysis is regarded as a promising option in water purification and sustainable environmental protection technologies [6, 7, 8, 9]. As for the fast destruction of toxic components in these photocatalytic devices, the fast generation and utilization of highly reactive oxygen species (ROS) play a crucial role [10, 11, 12]. However, it is fundamentally limited by the sluggish kinetics of charge separation and transport in conventional semiconductors [13]. The widespread adoption of conventional semiconductor photocatalysts (e.g., TiO_2_) is hampered by inherent limitations, including rapid charge‐carrier recombination, poor visible‐light absorption, and particle agglomeration [14]. Thus, the facile design of highly efficient photocatalysts is in high demand [15, 16, 17].

To transcend these limitations, the scientific pursuit has shifted toward designing multifunctional materials integrating enrichment and photocatalysis to enhance the ROS utilization rate [18]. By creating a unique microenvironment that is spatially confined and temporally synchronized, the behavior of reactants, intermediates, transition states, and products can be manipulated, ultimately achieving precise control over chemical reaction pathways. Nanoporous materials have shown significant advantages in photocatalytic reactions due to their tunable pore structure and high specific surface area, making them one of the most effective candidates for environmental remediation [19, 20]. Metal‐organic frameworks (MOFs) offer an ideal platform for this endeavor, not only for their porosity but also for their ability to create well‐defined, nanoconfined microenvironments that can modulate electronic structures [21, 22]. In particular, the nanoconfinement effect within MOF pores can enrich reactants, facilitate mass transfer, and modulate reaction pathways, leading to enhanced catalytic selectivity and efficiency [23, 24]. Despite these advantages, the photocatalytic performance of many pristine MOFs, such as those with symmetric Zr‐oxo clusters, remains suboptimal due to limited active sites and insufficient light‐harvesting capabilities [25, 26]. Ligand engineering and defect engineering offer powerful strategies to tailor the local coordination environment and create unsaturated metal sites, thereby activating the metal sites [27, 28]. In photosynthesis, chloroplasts use precise ‘antenna’ proteins to capture light energy spatially and transfer the energy to the reaction center in a very short time, preventing energy dissipation. In the thylakoid membranes of algae, two distinct photosystems (PSI and PSII) work in concert with mobile electron carriers like plastocyanin to achieve high energy conversion efficiency [29, 30]. This natural system separates the functions of light harvesting, charge separation, and electron transport across a confined membrane space, minimizing recombination losses [31]. Taking inspiration from plastocyanin, which bridges PSI and PSII in natural photosynthesis, we designed a bioinspired photocatalytic MOF by using TCPP as a connecting linker and introducing defects via ligand engineering. This design affords a confined system that synergistically combines ligand‐to‐cluster charge transfer (LCCT) with defect‐mediated active sites.

Herein, we report a bioinspired compartmentalized framework with a light‐harvesting antenna, constructed by confining a porphyrin derivative (TCPP) within the defect‐engineered matrix of MOF‐808. The precise size‐matching between TCPP and the defective MOF‐808 pores induces a spatial confinement effect that not only stabilizes the guest molecules but also fundamentally alters the electronic distribution of the hosts. Systematic experimental characterization and density functional theory (DFT) calculations reveal that TCPP acts as an efficient photosensitizer (analogous to PSI (Photosystem I)), injecting electrons into the defect‐rich Zr‐oxo clusters (functioning as PSII (Photosystem II)), and the ROS generated in the pore channel could be enriched and quickly consumed by pollutant adsorption at the defects. This process creates a compartmentalized porous material, which is drastically enhanced by the asymmetric coordination environment generated by the defect engineering. The resulting asymmetric electron density distribution across the Zr‐O‐TCPP interface significantly reduces the energy barrier for the formation of reactive oxygen species, steering the pathway toward singlet oxygen (^1^O_2_) generation and consumption.

## 2 Results and Discussion

### 2.1 Characterization

**FIGURE 1:**
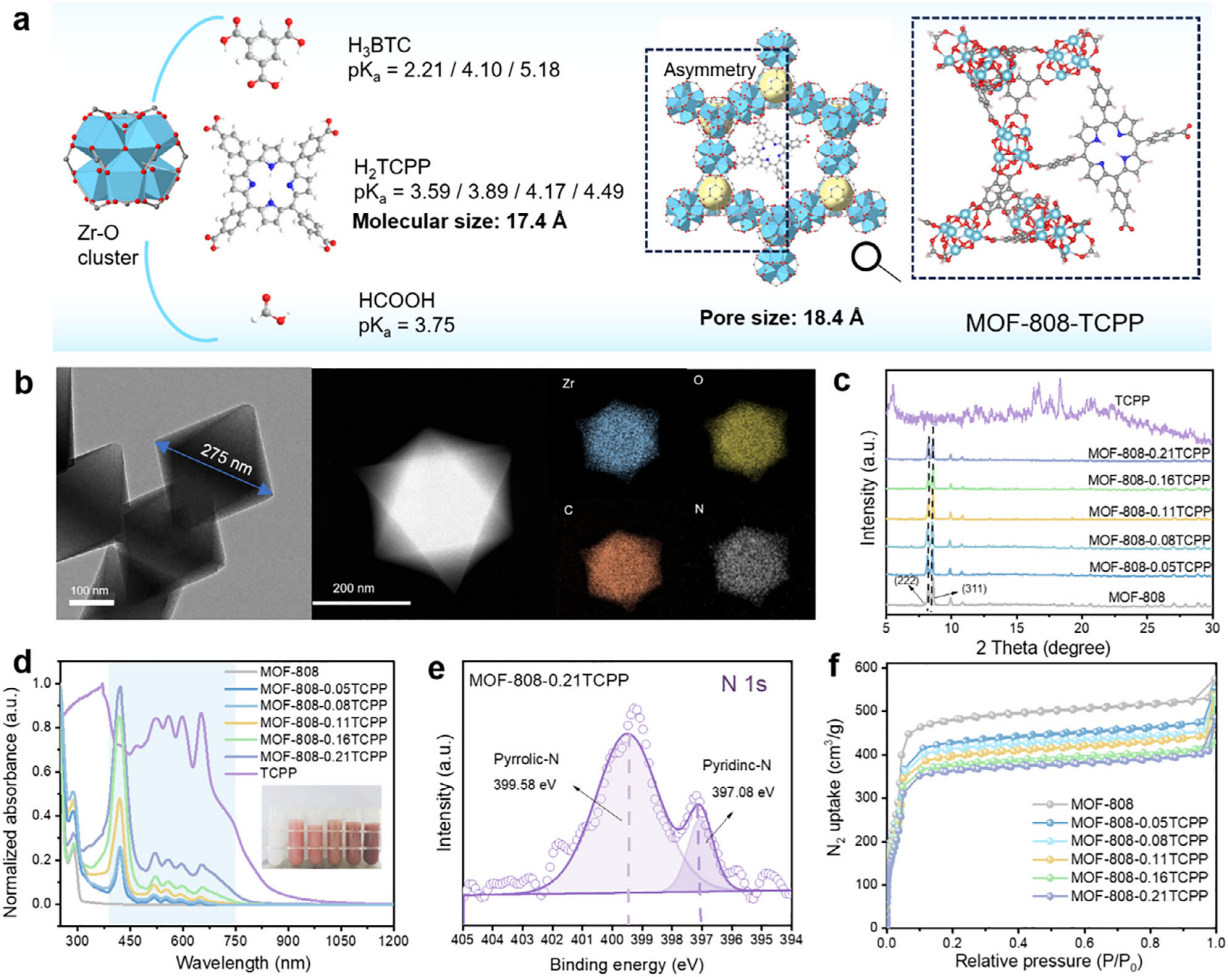
(a) The schematic diagram of TCPP@MOF‐808 synthesis. (b) The HRTEM and HADDF‐STEM of MOF‐808‐0.08TCPP. (c) PXRD profiles. (d) UV–vis DRS spectrum. (e) The N 1s spectrum from XPS analysis. (f) N_2_ adsorption and desorption curves.

The organic ligand with photosensitive properties, H_2_TCPP (1.747 × 1.748 × 0.578 nm), was chosen as the light‐harvesting antenna; then, porphyrin was encapsulated into the pores of MOFs via an in situ formation strategy (Figure 1a). During the formation of the metal‐based framework, the metal nodes, organic linkers, and porphyrin, the free‐based functional H_2_TCPP were entrapped inside the frame like a ship in a bottle. In the TCPP@MOFs, the porphyrinic MOFs were integrated as guest molecules via encapsulation in the pores through coordination with metal ions, like organic linkers. The formic acid (pK_a_ = 3.75) and carboxylate ligand (such as H_3_BTC, with pK_a_ values at 2.21, 4.10, and 5.18) coordinated with the Zr‐oxo cluster for the formation of mixed linker MOF‐808 [32, 33]. Through the pro‐coordinated formic acid to Zr‐oxo clusters, the functional linkers (meso‐Tetra(4‐carboxyphenyl) porphin, pK_a_ = 3.59, 3.89, 4.17, and 4.49) [34, 35, 36] were introduced into the framework, leading to the incomplete exchange. As a result, the structural defects would be generated. Thus, we envisioned that the structural defects could increase by adding more TCPP linkers with larger molecular sizes. Two possible coordination environments, including monodentate and bidentate patterns of TCPP@MOF‐808, have been considered, where the ‐COOH group in TCPP was coordinated onto Zr_6_O_8_ nodes. Among them, the most stable configuration was probably the bidentate model with two Zr clusters (E_ads_ = ‐11.09 kJ/mol) instead of the monodentate model (E_ads_ = ‐7.05 kJ/mol) (Figure S1). Therefore, the electron‐deficient center of the Zr atom on MOF‐808 was preferred to combine with the TCPP molecule in bidentate coordination. A series of samples of MOF‐808, MOF‐808‐0.05TCPP (mole ratio of TCPP/BTC is 0.05), MOF‐808‐0.08TCPP, MOF‐808‐0.11TCPP, MOF‐808‐0.16TCPP, and MOF‐808‐0.21TCPP were synthesized (Figure S2).

The synthesized materials were characterized by powder X‐ray diffraction (PXRD). After the incorporation of TCPP, the typical peaks corresponding to the (222) and (311) crystal plane structures of mixed linkers‐MOFs appeared at 8.3°and 8.8°, which were consistent with the pristine MOF‐808 (Figure 1c), indicating that the encapsulation of porphyrins in the interior of MOF‐808 (*spn* topology) showed negligible changes to the crystalline structure. The defective structure can be adjusted by changing the ratio of deprotonated H_2_TCPP, H_3_BTC, and formic acid through in situ formation methods [37]. Increasing amounts of TCPP were introduced and synthesized accordingly with (MOF‐808, MOF‐808‐0.05TCPP, MOF‐808‐0.08TCPP, MOF‐808‐0.11TCPP, MOF‐808‐0.16TCPP, MOF‐808‐0.21TCPP) with the TCPP: BTC calculated to be 0, 0.05, 0.08, 0.11, 0.16, and 0.21 (Table S1), respectively. To explore the chemical structure and functional groups, the Fourier transform infrared spectrum (FT‐IR) was analyzed (Figure S3 and Table S2). The wavenumbers 451 and 653 cm^−1^ corresponded to the stretching vibrations of the Zr‐O and Zr─O─Zr bonds, respectively, while 942 and 1109 cm^−1^ corresponded to the stretching vibrations of the C═C bond on the benzene ring. The wavenumber 1377 cm^−1^ corresponded to the symmetric stretching vibrations of the ‐COO in the BTC ligand. Peaks at 1444 and 1606 cm^−1^ corresponded to the symmetric and asymmetric stretching vibrations of the ─COOH bond, while 1656 cm^−1^ corresponded to the stretching vibration peak of ‐COO‐Zr. These absorption peaks confirmed the strong interaction, and the vibrational wavenumbers of the main functional groups in these TCPP@MOF‐808 shifted (653 to 660 cm^−1^ for Zr‐O‐Zr groups, 1656 to 1660 cm^−1^ for ‐COO‐Zr groups) [38, 39]. The electron clouds on the original MOF‐808 framework were rearranged and distributed based on the spatial hindrance, leading to an increased adsorption frequency of the corresponding functional groups in the FT‐IR spectrum [40]. Because of the coordination of oxygen and redistribution of electron clouds from TCPP ligands onto the Zr metals, the above increasing wavenumber shifted accordingly.

MOF‐808‐0.11TCPP showed regular octahedral morphology with an edge length of 275 nm (Figure 1b). According to the energy dispersive spectrometer mapping (EDS) (Figure 1b), the Zr, C, O, and N elements in MOF‐808‐0.11TCPP were uniformly distributed. The distribution of N (approximately 0.91%) confirmed the successful integration of TCPP. The existence of TCPP was further confirmed through UV–vis DRS spectroscopy (Figure 1d), where the TCPP chromophore had characteristic absorption bands at 420 nm and 500–750 nm, corresponding to the Soret band and four Q‐bands [41]. These materials retained specific quartet peaks attributed to TCPP, further proving that TCPP was successfully incorporated. As the dosages of TCPP increased, the UV–vis spectroscopy gradually showed a blue shift (2–7 nm), which was consistent with the infrared analysis. The X‐ray photoelectron spectroscopy (XPS) was analyzed to further study the compensated linkers and chemical structure of the metal cluster. By observing the full spectrum (Figure S4), the intensity of the N 1s peak at a binding energy of 400 eV increased significantly with the incorporation of TCPP, indicating that more TCPP was integrated into MOF‐808‐0.21TCPP. The high‐resolution scan was performed on the N 1s spectrum of MOF‐808‐0.21TCPP (Figure 1e), and peaks at 399.58 eV and 397.08 eV corresponded to C─N and pyridine C ═ N, respectively.

**FIGURE 2:**
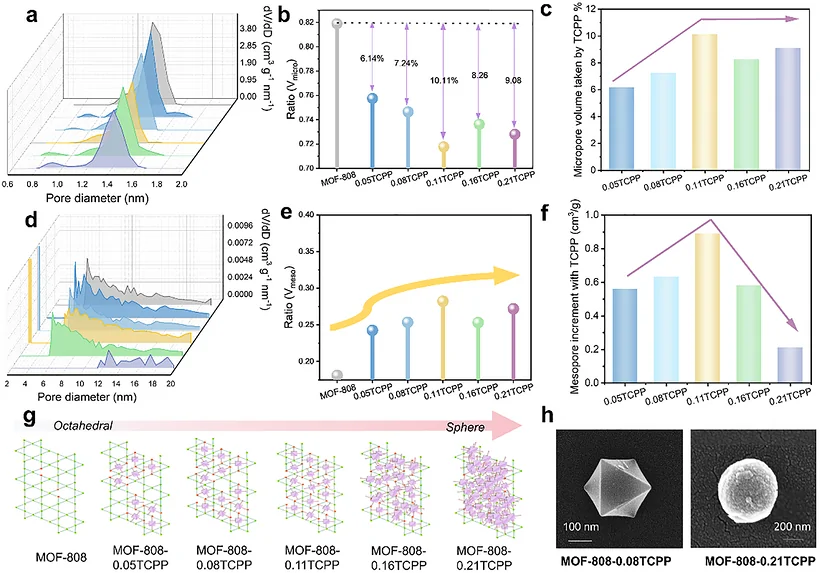
(a) The microporous distribution calculated by the QSDFT model. (b) The ratio of micropores with TCPP increases. (c) The micropore volume taken by TCPP. (d) The mesoporous distribution calculated by the QSDFT model. (e) The ratio of mesopores with TCPP increases. (f) Mesopores with TCPP. (g) The schematic diagram of morphology evolution with TCPP. (h) SEM of MOF‐808‐0.08TCPP and MOF‐808‐0.21TCPP.

The N_2_ adsorption‐desorption isotherm of the series MOF‐808 belongs to a type I isotherm (Figure 1f), indicating the encapsulation of TCPP within MOFs. The surface area gradually decreased with more TCPP incorporated (Table S3). It could be seen that the BET specific surface area of MOF‐808, MOF‐808‐0.05TCPP, MOF‐808‐0.08TCPP, MOF‐808‐0.11TCPP, MOF‐808‐0.16TCPP, and MOF‐808‐0.21TCPP were calculated to be 1606, 1325, 1312, 1283, 1231, and 1054 m^2^/g, respectively. The pore size distribution of TCPP@MOF‐808 was measured and simulated by the QSDFT model (Quenched solid density functional theory) (Figure S5). The major simulated pore channel of TCPP@MOF‐808 ranged from 1.3 to 1.5 nm (Figure 2a,d). The ratio of micropores with confined TCPP significantly decreased from 81.89% in MOF‐808 to 72.81% in MOF‐808‐0.21TCPP (Figure 2b), caused by the encapsulation of TCPP in the micropore channel of MOF‐808 [28, 42]. The ratio of micropore volume occupied by TCPP was calculated (Figure 2c), appearing at saturation in MOF‐808‐0.11TCPP because of the limitation of defect sites in the pore channel. As the amount of TCPP further increased, the redundant ligands preferred to coordinate at the surface of MOFs (Figure 2g,h) [43]. The ratio of mesoporous volume increased by 6.14%∼10.11% with TCPP incorporation, probably because the introduction of large‐sized ligands formed asymmetric structures, thus creating mesopores (Figure 2e). The minimum of microporous volume (V_meso_ = 0.717 cm^3^/g) and the maximum of mesoporous volume (V_meso_ = 0.282 cm^3^/g) can be observed at MOF‐808‐0.11TCPP (Figure 2f and Table S3). Therefore, the asymmetrical coordination led to an increase in mesopore volume with defect sites, and the pore size distribution confirmed the successful confinement of TCPP within the micropore channel. The scanning electron microscope (SEM) exhibited a clear octahedral shape of MOF‐808 with a smooth surface and uniform particle distribution. With increasing TCPP amount added, the morphology changed and became slightly rough (Figure S6). The original octahedral structure of MOF‐808 disappeared and was progressively transformed into a quasi‐spherical shape. When the TCPP/BTC ratio was less than 0.08, the crystal morphology still maintained a clear octahedral structure. As the ratio increased to 0.11, the initial octahedral edges significantly differed from the perfect octahedron. It should be noted that when the TCPP/BTC ratio reached 0.21, the cleaned octahedral shape was completely unobservable and presented a quasi‐spherical shape. Additionally, the particle size gradually decreased with an increasing ratio of TCPP (TCPP/BTC = 0.05 to 0.11). Thus, a small amount of TCPP in MOF‐808‐0.05TCPP, MOF‐808‐0.08TCPP, and MOF‐808‐0.11TCPP could generate localized defects. In comparison, when the TCPP ligands in the framework reached saturation in confined pores, bidentate coordination (H_2_TCPP) within the pore channel shifted to monodentate ligands coated on the surface.

**FIGURE 3:**
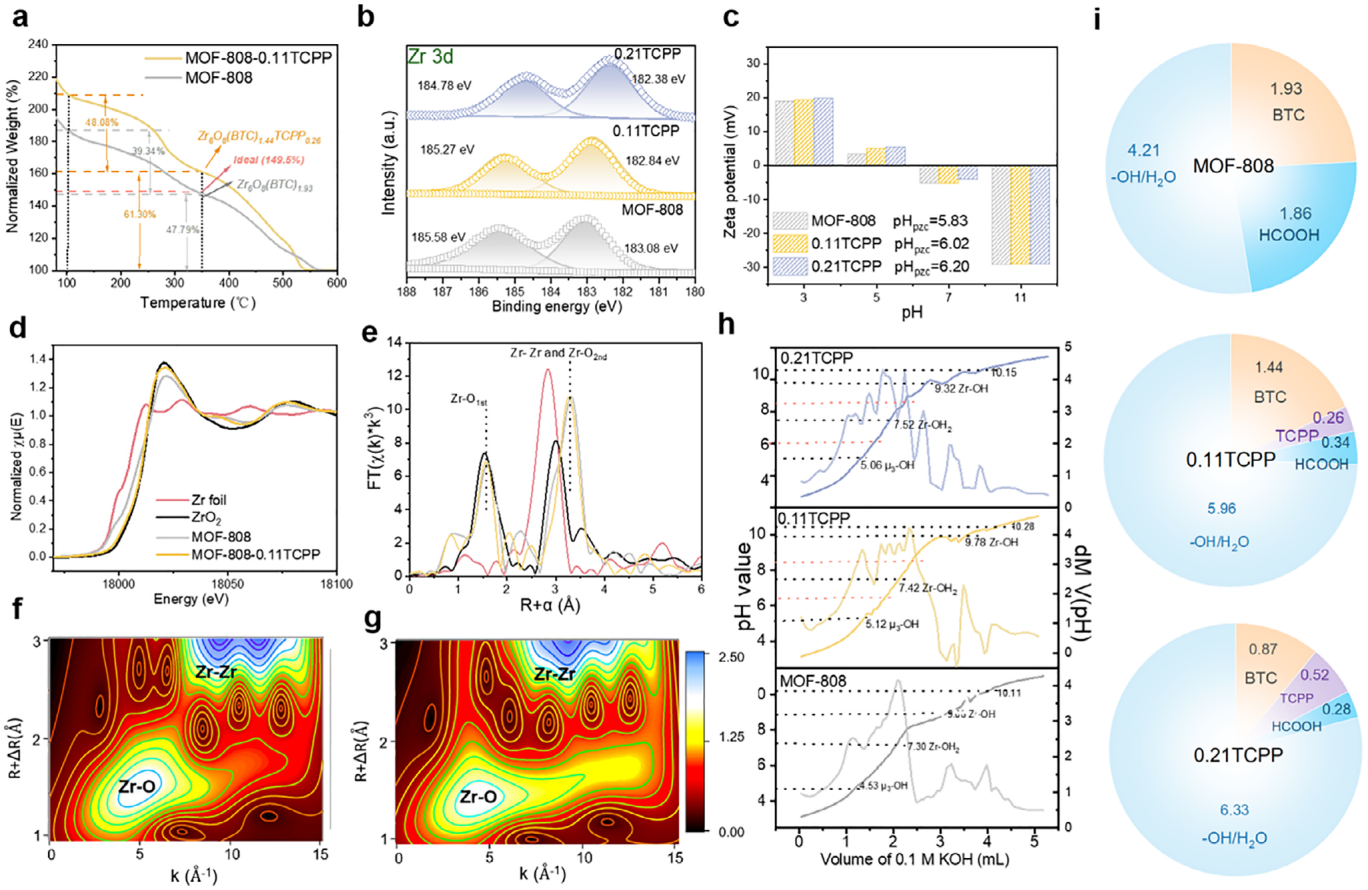
(a) Thermogravimetric curves and calculation results. (b) The Zr 3d spectrum of MOF‐808, MOF‐808‐0.11TCPP, and MOF‐808‐0.21TCPP. (c) Zeta potential of MOF‐808, MOF‐808‐0.11TCPP, and MOF‐808‐0.21TCPP. (d) Normalized XANES and (e) EXAFS spectra of MOF‐808 and MOF‐808‐0.11TCPP. (f) WT‐EXAFS of MOF‐808. (g) WT‐EXAFS of MOF‐808‐0.11TCPP. (h) Potentiometric acid‐base titration. (i) The coordination number of BTC, TCPP, HCOOH, and compensated ligands of MOF‐808, MOF‐808‐0.11TCPP, and MOF‐808‐0.21TCPP.

Confined TCPP in pores caused an asymmetric Zr‐O cluster, probably leading to an increasing number of defective sites [44]. The ratio of Zr, O, C, and N of TCPP@MOF‐808 was determined using semi‐quantitative elemental analysis (Figure S7 and Table S4). The atomic content of N in the MOF‐808‐0.05TCPP, MOF‐808‐0.08TCPP, MOF‐808‐0.11TCPP, MOF‐808‐0.16TCPP, and MOF‐808‐0.21TCPP gradually increased to 0.57%, 0.77%, 1.05%, 1.58%, and 2.06%, respectively. In comparison, the atom ratio of Zr in MOF‐808 (5.76%), MOF‐808‐0.05TCPP (5.63%), MOF‐808‐0.08TCPP (5.44%), MOF‐808‐0.11TCPP (5.23%), MOF‐808‐0.16TCPP (4.71%), and MOF‐808‐0.21TCPP (4.82%) decreased gradually. Therefore, the amount of TCPP in SBU could be deduced depending on the atomic ratio of Zr/N per unit in Zr_6_O_8_(BTC)_x_(TCPP)_y_, where y = 0.14, 0.19, 0.26, 0.42, and 0.52. The thermogravimetric curve (TG) was analyzed to quantitatively calculate the number of BTC ligands in defective TCPP@MOF‐808 (100°C–600°C) (Figure 3a and Figure S8). The first weight loss occurred at 100°C and 350°C, corresponding to the absence of HCOOH, ‐OH, and possibly bound water at defect sites [45]. This platform represented a solvent‐ and regulator‐free dehydroxylated MOF (Zr_6_O_9_(BTC)_2_, M_w_ = 1105.57 g/mol). With more TCPP added, weight loss in the first stage gradually increased from 39.34% to 56.58%, possibly due to the generation of defects and the removal of binding water and formate. The second platform was obtained after 350°C and represented ZrO_2_ (M_w_ = 739.34 g/mol), corresponding to the absence of ligands BTC (M_w_ = 210.14 g/mol) and TCPP (M_w_ = 790.77 g/mol). Similar to that in the first stage, the weight loss ratio increased from 47.79% to 72.32%, which is 47.79% weight loss of MOF‐808 corresponding to the BTC ligands connectivity of 1.93, which is approaching the theoretical connectivity of 2.00 (49.50% weight loss). Thus, the BTC ligands of MOF‐808, MOF‐808‐0.05TCPP, MOF‐808‐0.11TCPP, MOF‐808‐0.16TCPP, and MOF‐808‐0.21TCPP were quantitatively calculated to be 1.93, 1.84, 1.51, 1.44, 1.08, and 0.87, respectively (Table S5 and Figure S9). Thus, it further proved that the successful coordination of TCPP on the Zr‐oxo cluster and the presence of large amounts of defects compared to that of the pristine MOF. The peak of the XPS spectrum of O 1s of MOF‐808‐0.11TCPP at 529.78 eV represents the lattice oxygen corresponding to Zr‐O‐Zr, which showed a significant change compared to the defect‐free MOF‐808 (529.26 eV) (Figure S10) [46]. It could be caused by the sufficient coordination of TCPP with exposed terminal oxygen atoms in Zr‐oxo clusters. The peak at 531.28 eV of MOF‐808‐0.11TCPP represented the chemically adsorbed oxygen at the oxygen vacancy [47], where ‐OH /H_2_O was absorbed at the Zr‐O center on the MOF‐808‐0.11TCPP surface. Compared with the defect‐free MOF‐808 (531.09 eV), the peaks representing the oxygen vacancy in MOF‐808‐0.11TCPP and MOF‐808‐0.21TCPP (531.28 eV) shifted to higher binding energies. The binding energy at 531 eV (compensated group ‐OH/H_2_O in metal node) gradually increased in TCPP@MOF‐808 compared to pristine MOF‐808, indicating the replacement of TCPP and an increasing defect density. Also, the peak at 532.28 eV of MOF‐808‐0.11TCPP was attributed to oxygen from C‐O/C = O or formic acid, which showed a decreasing trend in comparison to MOF‐808. This result also proves that TCPP could gradually replace the coordinated formic acid. According to the XPS spectrum of Zr 3d (Figure 3b), two characteristic peaks of Zr 3d (185.58 eV and 183.08 eV) shifted toward lower binding energies (185.27 and 182.84 eV), indicating that the defects in MOF‐808‐0.11TCPP resulted in low coordination numbers [48]. Zeta potentials of MOF‐808, MOF‐808‐0.11TCPP, and MOF‐808‐0.21TCPP were measured over a wide pH range of 3–11 (Figure 3c). The isoelectric points of MOF‐808 (p*l* = 5.83), MOF‐808‐0.11TCPP (p*l* = 6.02), and MOF‐808‐0.21TCPP (p*l* = 6.20) increased obviously, probably because of the increasing density of structural defects.

The electronic and atomic structure of TCPP@MOF‐808 samples was further studied using X‐ray absorption spectroscopy (XAS). Normalized Zr K‐edge X‐ray absorption near‐edge structure (XANES) spectra of MOF‐808 and TCPP@MOF‐808 were evidence for the occurrence of Zr (IV) ions (Figure 3d and Figures S11 and S12). The quantitatively fitted results via EXAFS were evaluated to authenticate the local coordination configuration of Zr in both TCPP@MOF‐808 and MOF‐808. As shown in Figure 3e and Table S6, two main peaks in MOF‐808 and MOF‐808‐0.11TCPP indicated that the first shell of Zr‐O was at 2.18 Å ∼ 2.20 Å and the second coordination shell of Zr‐Zr and Zr‐O‐Zr scattering was at 2.69 Å ∼ 2.71 Å. The Zr‐O length shrank slightly with the incorporation of TCPP ligands (Figure S13). The coordination number of both Zr shells increased from 3.4/5.6 to 3.7/5.9, implying that the linker‐missing defects in the material led to the exposure of Zr atoms and formed coordination‐unsaturated sites, which were occupied by water and hydroxyl groups [33]. The atomic configurations of TCPP@MOF‐808 and MOF‐808 were further examined subsequently using Zr‐K‐edge wavelet transform (WT)‐EXAFS, indicating the significant variations in the Zr‐O coordination environment (Figure 3f,g).

MOF‐808 includes 6‐connected cluster (Zr_6_(µ_3_‐O)_4_(µ_3_‐OH)_4_(HCOO)_6_) and H_3_BTC linker. The pristine MOF‐808 had three types of protons (µ_3_‐OH, ‐OH_2_, and ‐OH), for which the acid‐base titrations were at 5.14, 7.55, 8.74, and 10.02 mL (KOH), corresponding to pK_a_ values at 3.6, 6.22, 8.23, and 9.12. The titration points of activated MOF‐808 were comparable to previous studies (4.53, 7.30, 9.06, and 10.11 mL for µ_3_‐OH, ‐OH_2_, and ‐OH, respectively) (Figure 3h). In comparison, the titration points of MOF‐808‐0.11TCPP were located at 5.12, 7.71, 9.78, and 10.28 mL, with larger KOH consumption. The changing profiles of titration curves indicated structural defects with increasing TCPP [49, 50]. In addition, 1,3,5‐benzenetricarboxylate (BTC) and formate salt concentrations were quantitatively analyzed using 1H NMR spectroscopy (Figure S14). Due to the known number of BTC calculated by TGA, the number of HCOOH could be deduced depending on the ratio of BTC/HCOOH based on 1H NMR. The peak area of BTC (7.95 ppm) and formate salts (8.18 ppm) in MOF‐808 was comparable (the ratio of BTC/HCOOH was 3.86/4.00). The peak area of formate gradually decreases with more TCPP until the peak area ratio decreases to around 1:4 [51, 52]. Overall, based on the XPS analysis, TGA calculation, as well as 1H NMR experiments, the coordination of 1,3,5‐benzenetricarboxylate (BTC), formate, as well as TCPP was semi‐quantitatively calculated (Figure 3i and Table S5 and Figure s15) [53, 54]. The accurate TCPP coordination was also analyzed using N content in XPS and TG curves. In summary, based on the above discussion, the TCPP probably replaced the position of formic acid ligands with smaller steric hindrance than BTC in the Zr‐O cluster.

### 2.2 Enrichment and Photocatalysis of Micropollutants

**FIGURE 4:**
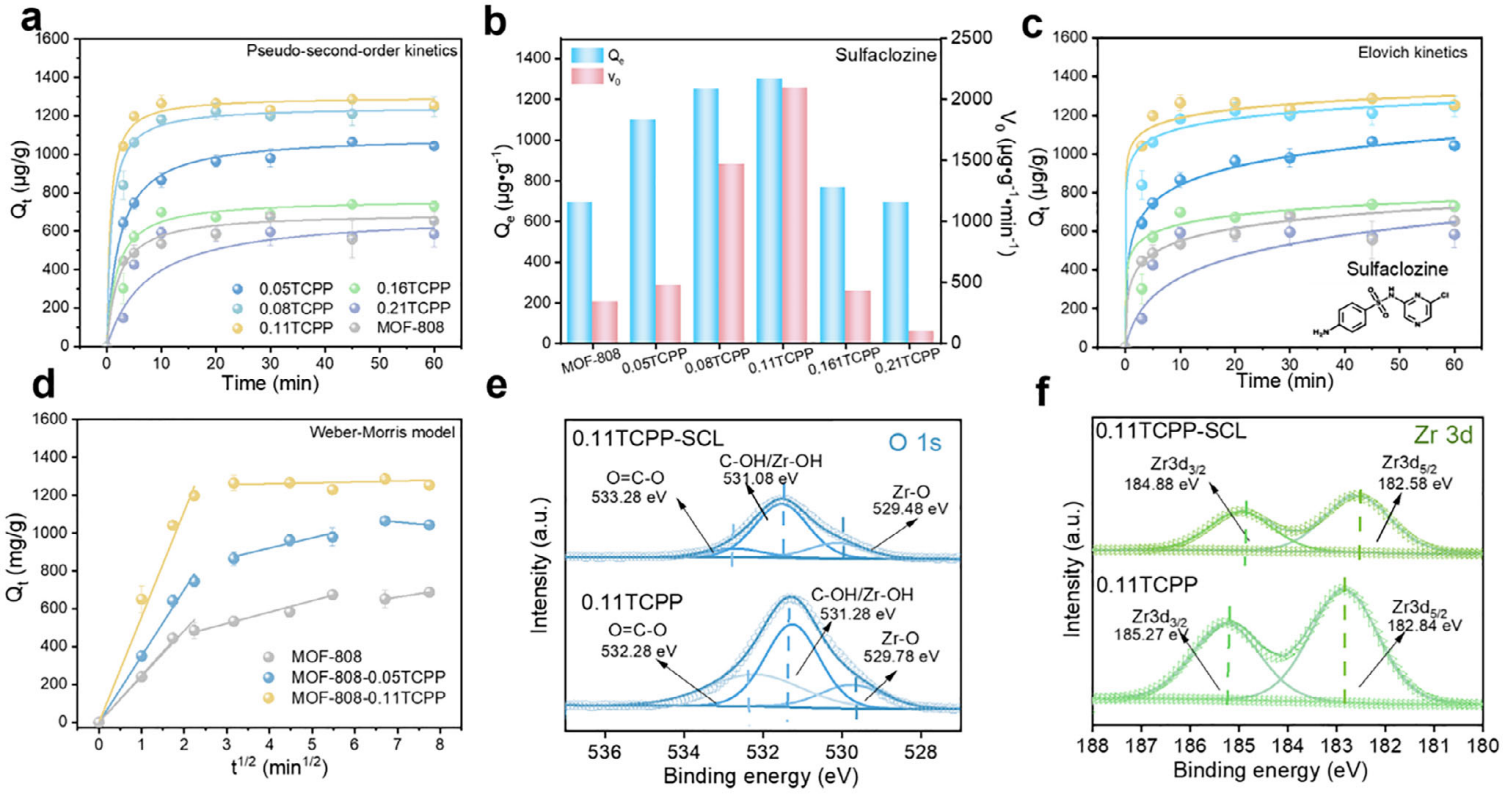
(a) The pseudo‐second‐order (PSO) kinetics of SCL. (b) The adsorption capacities (Q_e_) and initial rates (v_0_) of SCL over TCPP@MOF‐808 were calculated by the PSO model. (c) Elovich kinetics of SCL. (d) The Weber‐Morris model of SCL over TCPP@MOF‐808. (e) O 1s and (f) Zr 3d spectrum of MOF‐808‐0.11TCPP and MOF‐808‐0.11TCPP‐SCL. Reaction conditions: reaction volume of 50 mL, the initial concentration of SCL was 800 µg/L, and the catalyst dosage was 2 mg. Replicates (*n* = 3), error bars (mean ± SD), and statistical methods are detailed in the Experimental section and SI.

The adsorption kinetics of five kinds of pharmaceuticals at 800 µg/L, including nonsteroidal anti‐inflammatory drugs, clofibric acid (CA), ketoprofen (KP), diclofenac (DF), ibuprofen (IBU), and sulfaclozine (SCL) (Table S7), were fitted using three mathematical models, including the pseudo‐second‐order (PSO) model, Elovich, and the Weber‐Morris intraparticle diffusion model. The adsorption process of SCL (Figure S16) reached equilibrium within 60 min. The PSO kinetic model (Figure 4a and Table S8) was selected to describe the adsorption processes. The calculated adsorption capacities of SCL showed an order of MOF‐808‐0.11TCPP (1298.9 µg/g) >MOF‐808‐0.08TCPP (1248.5 µg/g) >MOF‐808‐0.05TCPP (1098.3 µg/g) >MOF‐808‐0.16TCPP (764.8 µg/g) >MOF‐808‐0.21TCPP (693.3 µg/g), which was consistent with the mesopore volume. The increasing adsorption capacities could be attributed to exposed defective sites. The optimal initial adsorption rate appeared over MOF‐808‐0.11TCPP (2092.3 µg/(g·min)), probably because of the higher mesopore volume (0.282 cm^3^/g) and proportion of V_mesopore_/V_total_ (28.22%) (Figure 4b). The adsorption profiles of ketoprofen (Figure S17), diclofenac (Figure S18), ibuprofen (Figure S19), and clofibric acid (Figure S20) showed a similar phenomenon. The maximum saturated adsorption amount and the optimal initial adsorption rate of ketoprofen (Table S9), diclofenac (Table S10), ibuprofen (Table S11), and clofibric acid (Table S12) both appeared in MOF‐808‐0.08TCPP and MOF‐808‐0.11TCPP, respectively (Figure S21). However, other PPCPs with high pK_a_, such as sulfamethoxazole (SMX), sulpiride (SP), carbamazepine (CBZ), and sulfaclozine (SCL), exhibited weak adsorption effects (Figure S22). The adsorption profiles of SCL and nonsteroidal anti‐inflammatory drugs (NSAIDs) over as‐prepared TCPP@MOF‐808 materials can be fitted highly relative to the Elovich model (R^2^>0.90), indicating that a chemical interaction could be one of the major mechanisms (Figure 4c). To further explore the advantages of reduced mass transfer resistance and adsorption mechanism, the Weber‐Morris intraparticle diffusion model was performed, where the Q_t_ value was correlated with t_1/2_. The fitted curve between Q_t_ and t_1/2_ for SCL adsorption can be divided into two different regions, probably because the surface diffusion and intra‐pore diffusion interaction lead to the adsorption mechanism. (Figure 4d and Figure S16). Usually, the first stage can be considered as external mass transfer, which represents the effect of the boundary layer. The data of k_i1_ of MOF‐808‐0.05TCPP, MOF‐808‐0.08TCPP, and MOF‐808‐0.11TCPP showed superiority over other materials, indicating that pollutants preferred to transfer from the boundary layer into the inner space. The value of k_i2_ obtained from the fitting calculation in the second stage was related to the mesoporous volume. The existence of mesopores could weaken the internal diffusion resistance and enhance the k_i2_ value of adsorption. The k_i2_ value of MOF‐808 (k_i2_ = 45.45 µg·g^−1^·min^−0.5^) showed that it had the highest internal diffusion resistance among the materials, and MOF‐808‐0.05TCPP, with more mesopores, showed the largest k_i2_ value (k_i2_ = 54.08 mg·g^−1^·min^−0.5^) (Table S8). However, the second phase disappeared in MOF‐808‐0.11TCPP due to the ultrafast adsorption rate. It could be observed that the C_i1_ value of the TCPP@MOF‐808 in the SCL adsorption approached zero, indicating that its internal diffusion controlled the initial adsorption rate. The C_i1_ of the Weber Morris model fitting of KP, DF, IBU, and CA for the series of materials also approached zero. Overall, the intra‐particle diffusion processes of CA, KP, DF, and IBU by adsorption of TCPP@MOF‐808 materials played an important role. After the adsorption equilibrium, the degradation profiles under the irradiation of solar light over the as‐prepared materials were studied.

The adsorption performance of SCL (pK_a_ = 2.0/5.5) and NSAIDs, such as CA (pK_a_ = 3.2) and KP (pK_a_ = 4.4), was found at pH 7.0 (Figure S23), where Zr‐OH/OH_2_ sites in adsorbents were positively charged, while SCL was mainly in a molecular state and NSAIDs were negatively charged. Therefore, electrostatic interaction has a relatively important influence on MOF‐808 adsorption. MOF‐808‐0.11TCPP and MOF‐808‐0.11TCPP‐SCL were selected to identify electrostatic interactions and *π–π* stacking interactions. In the XPS full spectrum, characteristic peaks of organic Cl and S from SCL could be observed in MOF‐808‐0.11TCPP after adsorption (Figure S24). Additionally, the peak intensity of N was significantly enhanced (Figure S25), proving that SCL had been successfully absorbed in the material. In the high‐resolution O 1s spectrum, the peak at 531.28 eV showed a slight decrease to 531.08 eV after adsorption, indicating that the SCL was absorbed near the oxygen vacancy (Figure 4e). In the Zr 3d spectrum, a pair of peaks at 185.27 and 182.84 eV was attributed to the Zr_6_ cluster in the MOF structure (Figure 4f), showing a shift toward 184.88 and 182.58 eV after absorbing SCL. The limited adsorption capacity of SCL resulted in a slight shift (less than 0.5 eV) in binding energy. The C1s spectrum of MOF‐808‐0.11TCPP exhibited three strong peaks at approximately 288.65, 286.37, and 284.87 eV, corresponding to the sp^2^ O─C═O and C═C groups in the trimesic acid ligand (Figure S26). After SCL adsorption, the characteristic peak representing the *π–π* component was observed at 292.3 eV, indicating the *π–π* stacking interaction between SCL and MOF‐808. Overall, the adsorption mechanism over MOF‐808‐0.11TCPP involved electrostatic and *π–π* stacking interactions.

**FIGURE 5:**
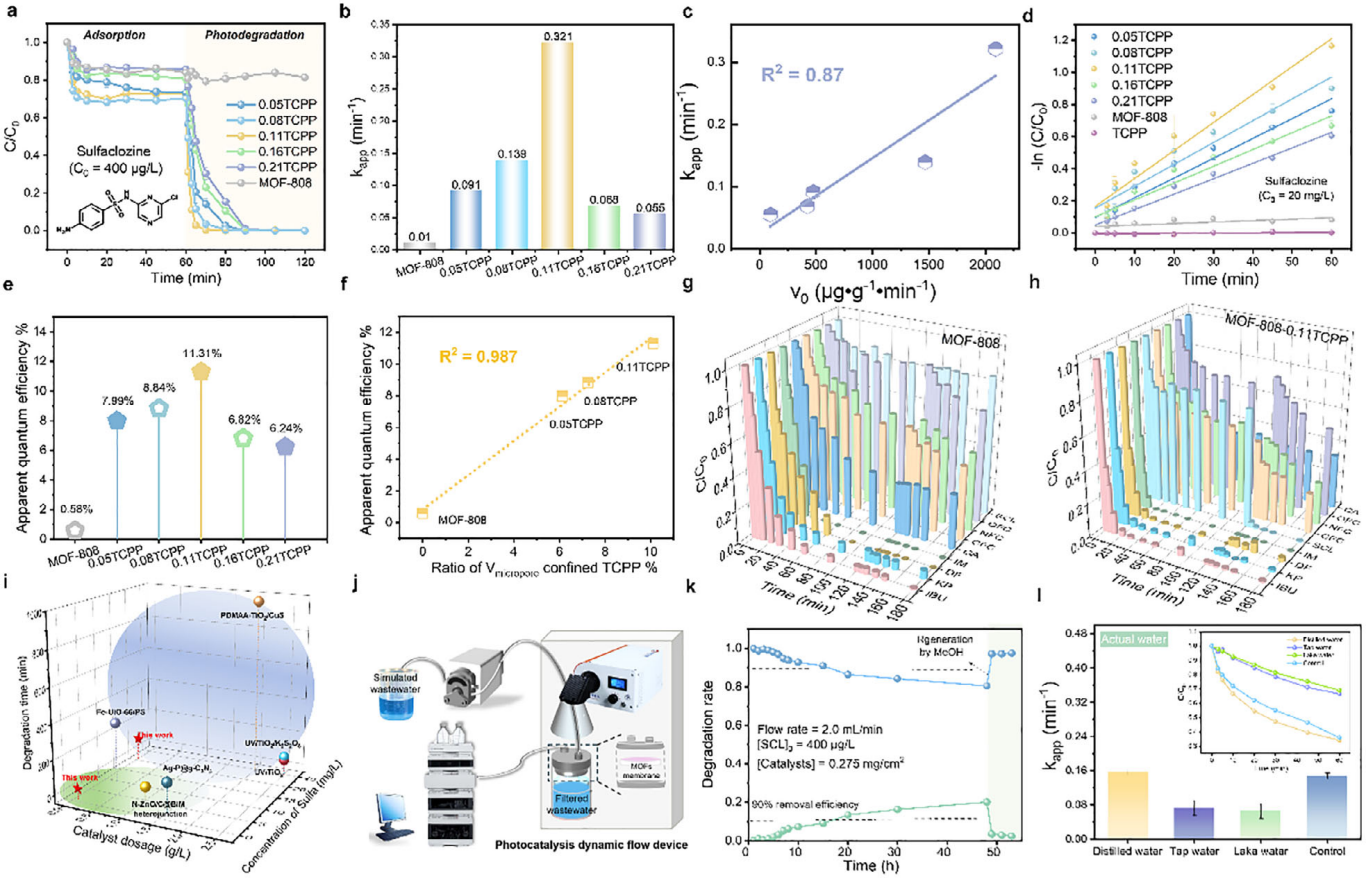
(a) Adsorption and photocatalysis of SCL (400 µg/L) and (b) the degradation rate constant of 400 µg/L of SCL. (c) The linear fitting of Q_e_ and v_0_ with k_app_. (d) The photodegradation kinetics of SCL (20 mg/L). (e) The apparent quantum efficiency of SCL with TCPP@MOF‐808. (f) The linear fitting of AQE with the amount of confined TCPP in the micropore. Adsorption (0∼120 min) and photocatalysis (120∼180 min) of multiple organic micropollutants by (g) MOF‐808 and (h) MOF‐808‐0.11TCPP. (i) The comparison of the performance. (j) The schematic diagram of the photocatalysis dynamic flow device with the MOF membrane. (k) The cycling performance of the MOF‐808‐0.11TCPP membrane. (l) The kinetics of SCL degradation in different water matrices. Reaction: 50 mL of reaction system, 0.05 g/L of catalysts, and a light intensity of 159.0 mW/cm^2^. Replicates (*n* = 3), error bars (mean ± SD), and statistical methods are detailed in the Experimental section and SI.

As shown in Figure 5a, the photocatalysis of SCL showed an apparent degradation. The low‐concentration SCL (400 µg/L) degraded completely (100%) over MOF‐808‐0.11TCPP within 30 min (Table S13). The maximum degradation rate constant was observed at MOF‐808‐0.11TCPP (k_app_ = 0.321 min^−1^), which was 2.3–5.9 times higher than that of other materials (Figure 5b and Figure S27). The relationship between adsorption and photodegradation was analyzed (Figure 5c). Notably, initial adsorption rates were well‐fitted with high correlation coefficients (R^2^ = 0.87), suggesting that adsorption performance exhibited a promoting influence on catalysis. The removal rate of high‐concentration SCL (20 mg/L) with MOF‐808‐0.11TCPP achieved 91.3% in 90 min (Figure S28 and Table S14), and MOF‐808‐0.11TCPP still maintained the best photocatalytic ability of the series materials (k_app_ = 0.0174 min^−1^) (Figure 5d). The apparent quantum efficiency (AQE) of series materials was calculated according to the reported work (Figure 5e) [40]. MOF‐808‐0.11TCPP exhibited a superior apparent quantum efficiency of 11.31%, significantly enhancing the SCL photocatalytic degradation efficiency under visible light irradiation (420 nm).

To correlate porosity with photocatalytic activity, we examined the pore architecture of the TCPP@MOF‐808 series and their apparent quantum efficiency (AQE), which can be found in Figure S29. When the TCPP molar ratio reached 0.11, the mesopore volume showed a positive correlation with the photocatalytic degradation rate constant (R^2^ = 0.99). However, further increasing the TCPP loading to 0.16 and 0.21 led to a sharp decline in both mesopore and micropore volumes, probably because excessive TCPP molecules within the limited space could reduce the accessible active sites. These observations suggest that the optimal TCPP loading (0.11) achieved a balance between maximized mesopore volume and accessible pore structure, which may contribute to the superior photocatalytic performance. Under conditions where TCPP exerted negligible influence on mass transfer, the AQE of MOF‐808‐0.05TCPP, MOF‐808‐0.08TCPP, and MOF‐808‐0.11TCPP showed a remarkably strong correlation with the TCPP‐occupied micropore volume (R^2^ = 0.999) (Figure 5f). However, because TCPP incorporation simultaneously affects defect density, light absorption, adsorption, and electronic structure, this correlation could be influenced by multiple mechanisms. Control experiments were designed to study the roles of confinement, defects, and adsorption (Figure S30). First, a physical mixture of free TCPP (equivalent to the amount in MOF‐808‐0.11TCPP) and pristine MOF‐808 showed negligible activity enhancement, indicating that simple coexistence has little influence on the high activity. Surface‐loaded TCPP and defect‐rich MOF‐808 were both synthesized, compared to MOF‐808‐0.11TCPP, indicating that neither surface immobilization nor defects alone could lead to higher performance. Furthermore, adsorption‐normalized experiments confirmed that MOF‐808‐0.11TCPP possesses higher intrinsic photodegradation activity per molecule. The control results are presented in Figure S30. In summary, while micropore volume (and TCPP confinement) show a strong correlation with activity, performance enhancement is not attributable to any single parameter. Due to the limited number of data points and the coupled effects of multiple factors, these correlations are presented as supportive observations rather than conclusive mechanistic evidence. Rather, the optimal activity of MOF‐808‐0.11TCPP reflects a synergistic synergy among confinement, defects, light absorption, and adsorption‐driven enrichment.

Compared to reported research, MOF‐808‐0.11TCPP in this work showed the highest photodegradation rate toward sulfaclozine (Figure 5g and Table S15). The turnover frequency of SCL (TOF, 3.21 × 10^−3^ min^−1^) over MOF‐808‐0.11TCPP is 32.1 and 36.90 times higher than those of MOF‐808 (0.10 × 10^−3^ min^−1^) and the conventional TiO_2_ photocatalyst (0.087 × 10^−3^ min^−1^), respectively [55]. Compared with other recently reported porphyrin‐MOFs and defect‐engineered Zr‐MOFs, MOF‐808‐0.11TCPP also exhibits great reaction kinetics for eliminating organic pollutants (Table S16), where TOF values were calculated and compared with recent work.

To further explore the performance of adsorption (dark control, 0∼120 min) and photocatalysis (light irradiation, 120∼180 min) of TCPP@MOF‐808, other 9 kinds of pharmaceuticals at 400 µg/L (ciprofloxacin (CFC), norfloxacin (NFC), ofloxacin (OFC), ibuprofen (IBU), diclofenac (DF), ketoprofen (KP), indomethacin (IM), clofibric acid (CA), and sulfaclozine (SCL)) were conducted (Figure 5h and Figure S31). As shown in Figure 5i, limited adsorption efficiency and photocatalytic ability over MOF‐808 were observed. After 120 min adsorption time, ibuprofen (IBU), diclofenac (DF), ketoprofen (KP), and indomethacin (IM) reached an adsorption percentage of over 98% with MOF‐808‐0.11TCPP, for which the photocatalysis performance was difficult to observe. Ciprofloxacin (CFC), norfloxacin (NFC), and ofloxacin (OFC) were degraded efficiently to 24.4%, 25.7%, and 54.1% within 60 min of light irradiation over MOF‐808‐0.11TCPP. Notably, SCL was completely degraded by MOF‐808‐0.11TCPP in 10 min of irradiation. This study indicates that the catalyst is particularly effective for removing SCL under the current conditions. The MOF‐808‐0.11TCPP/PVDF membrane was synthesized, and the continuous flow device was constructed (Figure 5j). The stability of TCPP@MOF‐808 was evaluated in a continuous flow setup using 0.5 L of sulfaclozine solution (400 µg/L) per cycle for 4 h and 10 cycles of continuous operation for 40 h. As shown in Figure 5j, degradation efficiency decreased by 21.07% in 40 h, probably because of the adsorption of organic intermediates within the framework (Figure S32a,b). The active framework can be regenerated to approximately 97% by comparing the surface area after the regeneration (1294 m^2^/g). The leaching of Zr ions was determined to be 29.198 µg/L after photocatalysis (Table S17). The PXRD results also showed that MOF‐808‐0.11TCPP maintained stability after reaction (Figure S32). XPS analysis of the catalyst before reaction and after regeneration showed only a 0.1∼0.2 eV shift in the Zr 3d and O 1s spectra, further confirming that the chemical states of Zr sites were not altered and the deactivation in the membrane can be regenerated (Figure S32).

To assess the practical application, the degradation performance of SCL with different catalyst dosages, initial concentrations, pH levels, co‐existing substances, and water matrix was performed (Table S18 and Figure S33). It could be observed that SCL degradation decreased from 0.150 to 0.053 min^−1^ in lake water and 0.060 min^−1^ in tap water. The inhibition in the actual water matrix might be caused by the co‐existing substances, whereas MOF‐808‐0.11TCPP still kept an effective photo‐degradation kinetic on SCL in the actual water matrix (Figure 5l). The SCL degradation rate increased with higher catalyst dosage. The optimal pH condition was in a neutral environment.

### 2.3 The Generation of ROS

**FIGURE 6:**
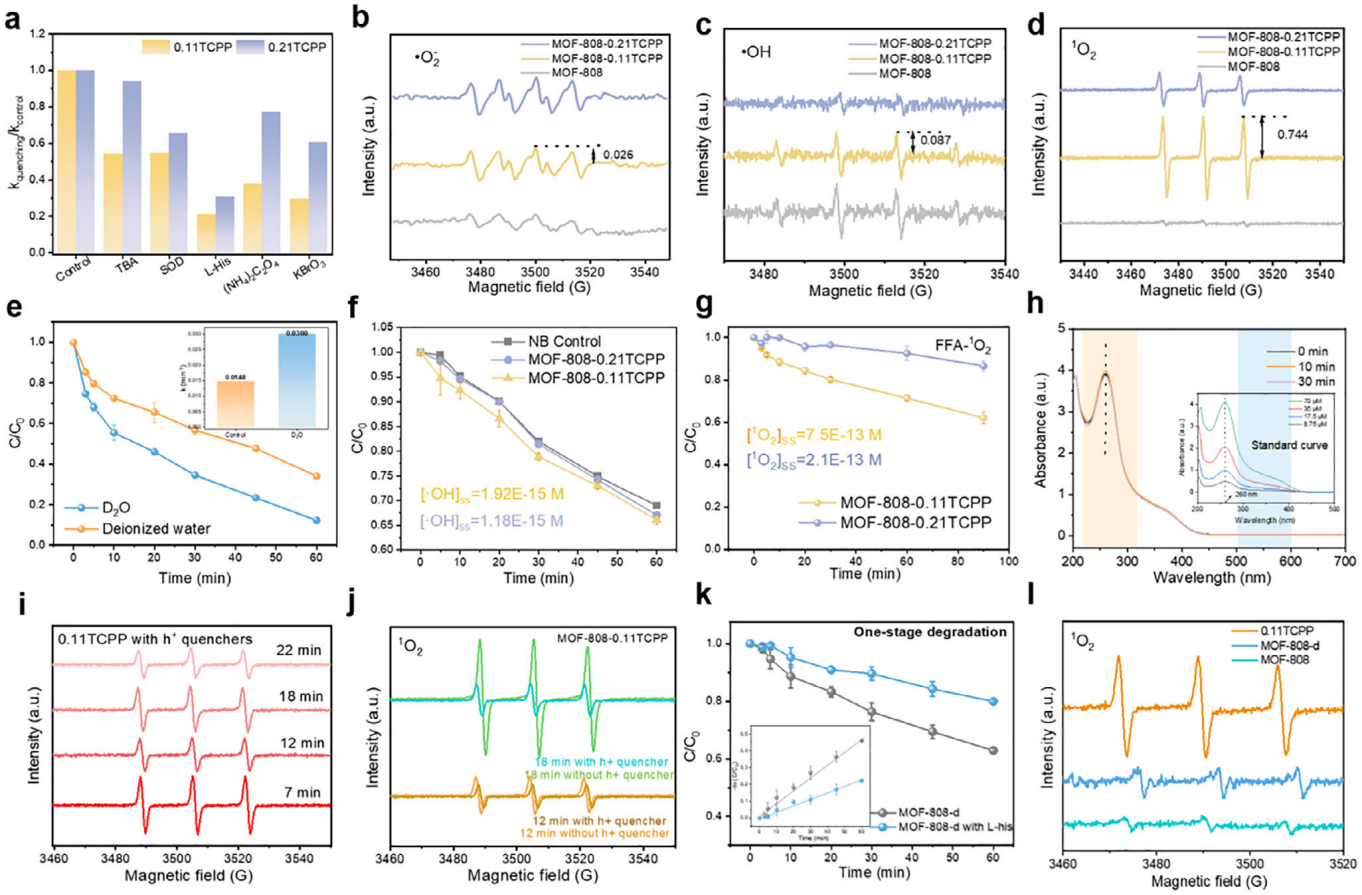
(a) Quenching result of MOF‐808‐0.11TCPP and MOF‐808‐0.21TCPP. ESR spectra of (b) superoxide anions, (c) hydroxyl radicals, and (d) singlet oxygen. (e) Photodegradation of SCL by MOF‐808‐0.11TCPP with 2 M D_2_O. The quantitative probes of (f) hydroxyl radicals with NB, (g) the quantitative probes of singlet oxygen with FFA, and (h) the UV–vis spectra of NBT (O_2_^•−^ probe test) in MOF‐808‐0.11TCPP systems. (i) ESR spectra of ^1^O_2_ with h^+^ quenchers of MOF‐808‐0.11TCPP at different times. (j) ESR spectra of ^1^O_2_ of MOF‐808‐0.11TCPP and MOF‐808‐0.11TCPP with h^+^ quenchers at 12 min and 18 min. (k) The photodegradation by MOF‐808‐d and MOF‐808‐d with L‐His. (l) The ESR signal of singlet oxygen of MOF‐808, MOF‐808‐d, and MOF‐808‐0.11TCPP. Reaction: 50 mL of reaction system, 0.05 g/L of catalysts, and a light intensity of 159.0 mW/cm^2^. Replicates (*n* = 3), error bars (mean ± SD), and statistical methods are detailed in the Experimental section and SI.

ESR spectra and quenching experiments were conducted to determine ROS formation. Tert‐butyl alcohol (TBA), superoxide dismutase (SOD), and L‐Histidine were selected to act as the scavengers for •OH, O_2_^•−^, and ^1^O_2_ (Figure 6a and Figure S34). After L‐His was added, the SCL degradation percentage over MOF‐808‐0.11TCPP and MOF‐808‐0.21TCPP decreased to 17.3% and 15.4%, respectively, indicating the crucial role of ^1^O_2_, which is a non‐radical pathway during the degradation process. In comparison, the degradation of SCL decreased to 44.9% and 25.7% over MOF‐808‐0.11TCPP and MOF‐808‐0.21TCPP, showing that the O_2_^•−^ also played an important role during photocatalysis. KBrO_3_ and (NH_4_)_2_C_2_O_4_ were used as the quenching agents for photogenerated e and h^+^, showing that these species also contributed to the degradation. In comparison, •OH provided a slight contribution to the degradation. In situ electron spin resonance (ESR) spectra of MOF‐808, MOF‐808‐0.11TCPP, and MOF‐808‐0.21TCPP under visible light irradiation were acquired. The signal intensity of DMPO‐O_2_^•−^ was slightly enhanced in MOF‐808‐0.11TCPP and MOF‐808‐0.21TCPP compared with MOF‐808, with an increase from 0.63 × 10^−3^ to 0.026 (Figure 6b). The DMPO‐•OH was detected in the TCPP@MOF‐808 system (Figure 6c), and the signal of TEMP‐^1^O_2_ significantly increased from 0.052 to 0.744 (14 times higher) with TCPP introduction (Figure 6d). During this process, TCPP could transfer electrons to the conduction band, and oxygen vacancies could trap electrons during the LCCT process, thereby improving the signal of TCPP@MOF‐808. D_2_O was added to evaluate the dominant role of ^1^O_2_ [56]. 2 mol of D_2_O were added to the photocatalyst system, since the D_2_O was proven to prolong the lifetime of ^1^O_2_, and SCL was dissolved in D_2_O/H_2_O with the same operational parameters. During the D_2_O system, the photocatalytic kinetics of SCL increased to 0.030 min^−1^, which was 2.04 times higher than that of the pristine system (Figure 6e). Thus, these phenomena further prove the significant role of ^1^O_2_.

To detect the steady‐state concentrations of ROS in this system, nitrobenzene (NB), tetrazolium blue chloride (NBT), and furfuryl alcohol (FFA) were selected as quantitative probes. The steady‐state concentration of •OH in the MOF‐808‐0.11TCPP and MOF‐808‐0.21TCPP systems was calculated to be 1.92 × 10^−15^ and 1.18 × 10^−15 ^M (Figure 6f). The steady‐state concentration of ^1^O_2_ in the MOF‐808‐0.11TCPP system was 7.5 × 10^−13^ M with FFA (Figure 6g), which was 3.57 times that of the MOF‐808‐0.21TCPP system. The [•OH]_ss_ in these systems were two orders of magnitude lower than [^1^O_2_]_ss_. Therefore, it can be determined that ^1^O_2_ was the main active species for degrading SCL, and electrons, holes, hydroxyl, and superoxide anions were all involved in the degradation process (Table S19). The NBT could be reduced to monoformaldehyde by O_2_^•−^, which had the maximum absorption at 530 nm in the UV–vis spectrum (Figure 6h). There was no peak of mono formaldehyde after the reaction, which contradicted the ESR and SOD quenching results, possibly because the concentration of O_2_^•−^ was quite low, resulting in the low solubility of NBT degradation products that might still adsorb in the material. To further explore the contribution of major reactive species, we performed additional scavenging experiments using p‐benzoquinone (p‐BQ), which was recently recognized as O_2_^•−^ scavenger. As shown in Figure S36, the addition of p‐BQ significantly suppressed the photocatalytic degradation of SCL, indicating that O_2_^•−^ indeed participated in the reaction. Together with the ESR measurements, the p‐BQ quenching results provided consistent evidence that O_2_^•−^ is generated as an intermediate species and subsequently contributes to the formation of ^1^O_2_ [57].

The ESR signals were recorded after adding hole inhibitors at different times (Figure 6i). Results showed that the intensity of characteristic peaks decreased gradually at 7–12 min and tended to remain stable at 12–18 min. Two groups of ESR analyses were performed to further evaluate the formation of ^1^O_2_ over MOF‐808‐0.11TCPP (with and without the existence of hole inhibitors) at 12–18 min (Figure 6j). Results showed that the peak intensities were similar, and no obvious decreasing trend could be observed. Thus, the formation of ^1^O_2_ by h^+^ could be one of the major mechanisms. A defect‐engineered MOF‐808 sample without TCPP (MOF‐808‐d) was also prepared, showing markedly lower ^1^O_2_ production and diminished SCL degradation activity (Figure 6k). By contrast, the strong TEMP‐^1^O_2_ signal of MOF‐808‐0.11TCPP reflects a synergistic cooperation between defect engineering and LCCT (Figure 6l).

### 2.4 The Electron Transfer Pathway

**FIGURE 7:**
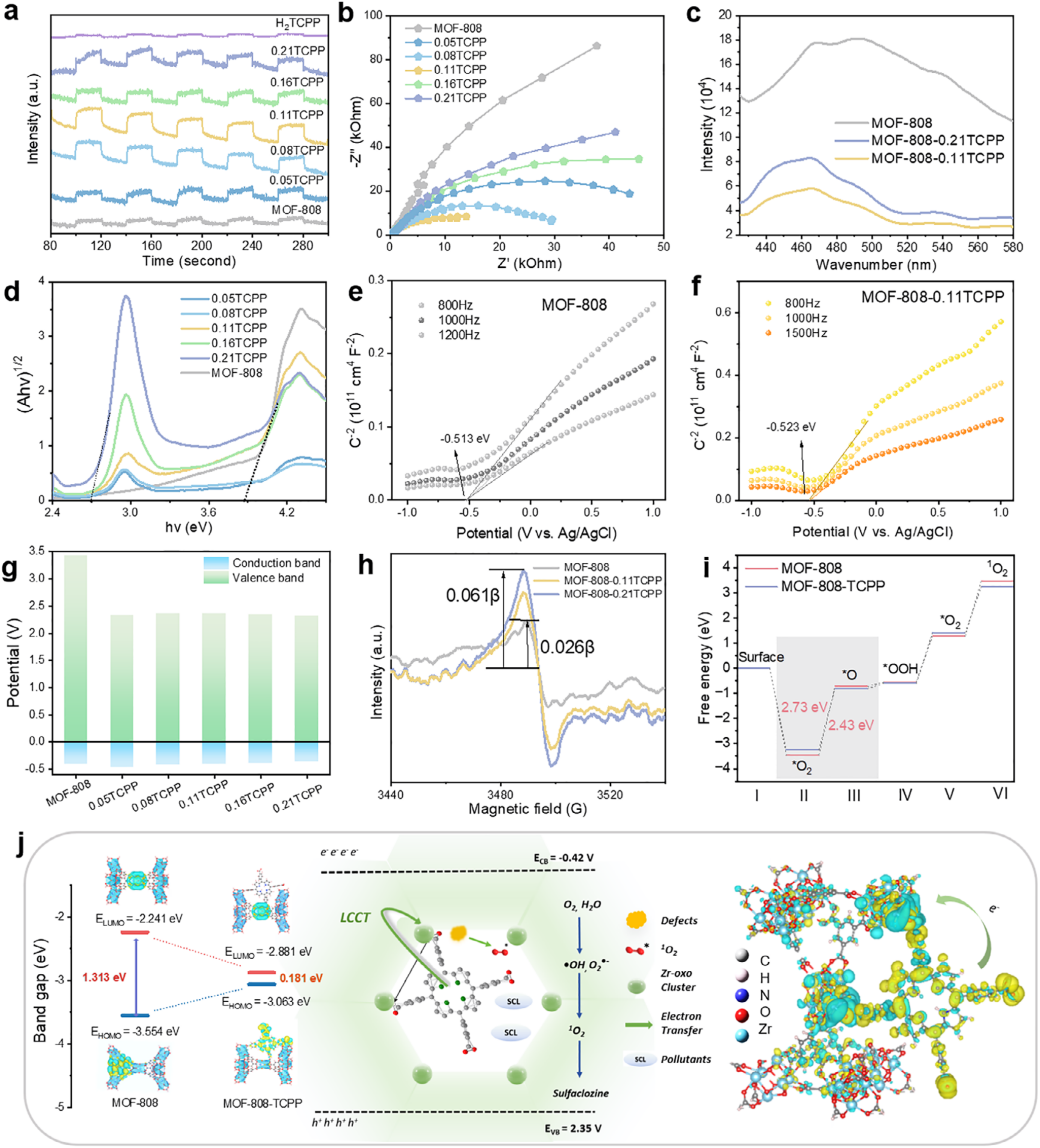
(a) The photocurrent response and (b) the EIS curves of TCPP@MOF‐808. (c) The PL spectra of MOF‐808, MOF‐808‐0.11TCPP, and MOF‐808‐0.21TCPP. (d) Tauc curves of TCPP@MOF‐808 by UV–vis DRS. Mott‐Schottky plots of (e) MOF‐808 and (f) MOF‐808‐0.11TCPP. (g) The calculated conduction band and valence band. (h) The ESR signal of MOF‐808‐TCPP. (i) Free energy of oxygen adsorption and activation process of MOF‐808 and MOF‐808‐0.11TCPP. (j) The HOMO and LUMO of MOF‐808 and MOF‐808‐TCPP, the charge density difference, and the schematic diagram of electron transfer.

MOF‐808‐0.11TCPP exhibited the highest photocurrent intensity, approximately 4.04 times that of pristine MOF‐808 (Figure 7a). Electrochemical impedance spectroscopy (EIS) revealed an order of charge‐transfer resistance following MOF‐808>MOF‐808‐0.21TCPP >MOF‐808‐0.16TCPP >MOF‐808‐0.05TCPP >MOF‐808‐0.08TCPP >MOF‐808‐0.11TCPP (Figure 7b), indicating that the combination of defects and TCPP incorporation synergistically enhances charge separation efficiency.

MOF‐808‐0.11TCPP exhibited the weakest photoluminescence (PL) emission intensity at an excitation wavelength of 390 nm (Figure 7c), indicating the most effective suppression of electron–hole recombination. This observation suggests that TCPP incorporation facilitates charge transfer and separation within the photocatalytic system. In addition, Mott–Schottky analysis and UV–vis diffuse reflectance spectroscopy (DRS) were performed to further elucidate the electronic band structure (Figure 7d). The bandgap energies (E_g_) of the TCPP@MOF‐808 series were determined using the Kubelka–Munk function. The corresponding Tauc plots revealed a gradual narrowing of E_g_ from 3.83 to 2.69 eV with increasing TCPP loading (Figures 7e,f and Figure S37). The conduction band (CB) potentials were derived from Mott–Schottky measurements, while the valence band (VB) potentials were calculated by combining the Mott–Schottky and Tauc results (Table S20). As summarized in Figure 7g, the CB edge of TCPP@MOF‐808 ranged from −0.47 to −0.37 V, and the VB edge ranged from 2.32 to 2.35 V (vs. NHE). These values are sufficiently negative to reduce O_2_ to superoxide radicals (E(O_2_/O_2_•^−^) = −0.33 V vs. NHE) [58], suggesting thermodynamic feasibility for O_2_•^−^ generation.

ESR analysis (Figure 7h) revealed an oxygen‐vacancy‐associated signal at g = 1.95 for TCPP@MOF‐808, corresponding to Zr(IV)‐to‐Zr(III) conversion upon LCCT enhancement [59, 60, 61]. The signal intensity was 2.3‐fold higher for MOF‐808‐0.21TCPP than for pristine MOF‐808. The electron transfer mechanism is depicted in Figure S38, where photoexcited electrons migrate from TCPP to the Zr–oxo cluster, further supported by charge density difference analysis (Figure 7i). Charge density difference analysis revealed that electron accumulation is concentrated on the TCPP ligands, whereas electron depletion (blue region) occurs at the Zr–O vacancies created by defect engineering (Figure 7i). This asymmetric charge distribution suggests that TCPP incorporation promotes charge separation. Furthermore, the rigid pore walls enforce a fixed orientation between the TCPP ligands and the Zr–oxo clusters, minimizing the electron transfer distance and providing a direct, low‐resistance pathway for photoexcited electrons. We further attribute the enhanced ROS generation to the synergistic effects of spatial confinement and electronic modulation. The confined microenvironment suppresses energy loss and extends the charge‐separated state lifetime, while the incorporation of TCPP and defects significantly alters the frontier orbital landscape. Specifically, the HOMO of MOF‐808‐0.11TCPP is predominantly localized on the TCPP ligands rather than the MOF‐808 framework (Figure 7j), accompanied by a narrowing of the bandgap from 1.313 eV (pristine MOF‐808) to 0.181 eV (TCPP@MOF‐808). This asymmetric charge distribution at the Zr–O–TCPP interface lowers the energy barrier for ROS production and favors the selective generation of ^1^O_2_. The current DFT calculations do not fully account for the aqueous environment, the actual defect distribution, or the explicit solvent effects. Therefore, the DFT results are used as a qualitative guide to understand the electronic structure trends rather than as definitive proof of the reaction mechanism.

The first five steps (I→V) corresponded to the O_2_ activation from *O_2_ to *O, *OOH, *O_2,_ and ^1^O_2_ using MOF‐808 (red lines) and TCPP@MOF‐808 (blue lines), respectively. As can be observed, the O_2_ adsorption free energy of TCPP@MOF‐808 (−3.24 eV) is slightly higher than that of MOF‐808 (red lines) (−3.46 eV). The lower adsorption energy between the Zr─O bond (*O_2_ to *O) decreased the activation barrier for the desorption and the following activation step to generate *O, *OOH, *O_2,_ and ^1^O_2._ The lower energy barrier (*O_2_ to *O) at the rate‐determining step (II→III) for O_2_ activation on MOF‐808‐TCPP (2.43 eV) favors the generation of ^1^O_2_ compared with that of MOF‐808 (2.73 eV), indicating the designed system facilitates the reactive oxygen generation (free energy decreased from 2.19 to 1.83 eV for the generation *O_2_→^1^O_2_). This process creates a directional LCCT pathway, which is drastically enhanced by the asymmetric coordination environment generated by the defects [54, 62].

## 3 Conclusion

A bioinspired photocatalyst has been developed based on the integration of a light‐harvesting porphyrin ligand (TCPP) within a defective Zr‐oxo cluster framework (MOF‐808). The rational design of components and ordered pore structures creates a nano‐confined environment that promotes charge separation and electron transfer from TCPP to the Zr‐oxo nodes, as evidenced by PL quenching, enhanced photocurrent, and reduced EIS resistance. This spatial organization leads to significantly improved photocatalytic degradation activity. While the system exhibits functional analogies to natural photosynthetic systems in terms of light harvesting and catalytic site separation, it does not replicate the full complexity of dual‐photosystem electron transport chains. Instead, it serves as a simplified bioinspired model for understanding charge‐transfer dynamics in confined MOF architectures. The TCPP‐based photocatalyst achieves an outstanding apparent quantum yield of 11.31% for the efficient degradation of micropollutants with high environmental risks, with a turnover frequency (3.21 × 10^−3^ min^−1^) that is 32.1 and 36.9 times higher than those of the pristine framework and conventional TiO_2_, respectively. Meanwhile, the specific Ligand‐to‐Cluster Charge Transfer (LCCT) mechanism, which functions as an “electron highway,” was significantly improved. The resulting asymmetric electron density distribution across the Zr‐O‐TCPP interface significantly reduces the energy barrier for the formation of reactive oxygen species, steering the pathway toward singlet oxygen (^1^O_2_) generation and consumption.

## 4 Experimental Section

### 4.1 Chemicals

All chemicals in this study were used as received without further purification. Zirconium tetrachloride (ZrCl_57_, >98%), 1,3,5‐benzenetricarboxylic acid (H_3_BTC, ≥98%), N, N‐dimethylformamide (DMF, 99%), acetone (>99%) were purchased from Shanghai Macklin Biochemical. 4,4',4'',4'''‐(21H, 23H‐porphine‐5,10,15,20‐tetrayl) tetrakis‐benzoic acid (H_2_TCPP, >99%) was from Meryer (Shanghai) Chemical Technology Co. Ltd. Formic acid (>99%) was obtained from Shanghai Aladdin Industrial Corporation. All the pharmaceuticals were purchased from Shanghai Yuanye Biotechnology using HPLC‐grade solvents, including sulfaclozine, clofibric acid, ketoprofen, ibuprofen, diclofenac, etc.

### 4.2 Preparation of MOF‐808

MOF‐808 was synthesized by following a slightly modified reported procedure. 0.75 mmol of ZrCl_4_ and 0.25 mmol of H_3_BTC were dissolved in 8 mL of DMF and 8 mL of formic acid in a 50 mL glass vial with a screw cap. The suspension was then sonicated for 10 min and heated at 120°C for 24 h. After cooling to room temperature, the obtained sample was washed with DMF and acetone, each for 2 days, to remove impurities. Finally, the obtained MOF‐808 powder was dried and activated in a vacuum at 80°C for 10 h.

### 4.3 Preparation of TCPP@MOF‐808

To synthesize the series of TCPP@MOF‐808, different amounts of H_2_TCPP were added during the synthesis process (Table S1), with the molar ratios of H_2_TCPP and H_3_BTC being 0.05, 0.08, 0.11, 0.16, and 0.21, respectively. 0.75 mmol of ZrCl_4_, 0.25 mmol of H_3_BTC, and 0.0267 mmol (40 mg) of H_2_TCPP (TCPP/BTC = 0.11) were dissolved in 8 mL of DMF and 8 mL of formic acid in a 50 mL glass vial with a screw cap. Then the suspension turned dark green after 10 min of ultrasonic treatment and was heated at 120°C for 24 h. After cooling to room temperature, the obtained sample was washed with DMF and acetone, each for 2 days. Finally, the pink MOF‐808‐0.11TCPP powder was activated in a vacuum at 80°C for 10 h.

### 4.4 Characterization

The characteristics of the material were analyzed by powder X‐ray diffraction (PXRD, Mini Flex 600, Rigaku Corporation, Japan) and Fourier transform infrared spectroscopy (FT‐IR, ALPHA, Bruker, Germany). The morphology of the material was evaluated by transmission electron microscopy (TEM, JEOL JEM‐F200, Japan), scanning electron microscopy (SEM, JSM‐7500F Hitachi, Japan), and an energy dispersive spectrometer (EDS). The surface area and pore size distribution were tested by N_2_ adsorption (KuboX1000, Beijing Biotech Electronics Co., Ltd.). The defect structure of the synthesized materials was described using Zeta potential (Malvern Zetasizer Nano ZS ZEN3600, UK), thermogravimetric analysis (TGA, TG‐DTA 8122, Rigaku, Japan), H Nuclear Magnetic Resonance Spectra (1H NMR, Bruker 400 MHz, Germany), X‐ray photoelectron spectroscopy (XPS, Thermo Scientific K‐Alpha, USA), electron spin resonance (ESR, Bruker EMX plus, Germany) measurements, and XANES spectra measurements. The photoelectric properties of the material were comprehensively investigated by conducting solid‐state ultraviolet‐visible diffuse reflectance spectroscopy (UV–vis DRS, UV‐2600 spectrometer, SHIMADZU, Japan), electrochemical impedance spectroscopy, photocurrent measurements, and Mott‐Schottky curves (CHI‐660E, China). The leaching concentration of Zr ions was measured using an inductively coupled plasma‐mass spectrometer (ICP‐MS, PE NexION 350X, America).

### 4.5 Adsorption and Photodegradation Experiment

Removal of PPCPs in simulated experiments of various environmental concentrations was tested. A high concentration solution (20 mg/L) of 13 types of PPCPs was first prepared, including non‐steroidal anti‐inflammatory drugs (NSAIDs), diclofenac (DF), ketoprofen (KP), indomethacin (IM), ibuprofen (IBU), and chloramphenicol (CA), sulfonamide drugs such as sulfaclozine (SCL) and sulfamethoxazole (SMX), quinolone drugs (norfloxacin (NFC), ofloxacin (OFC), ciprofloxacin (CFC)), and others (carbamazepine (CBZ) and sulpiride (SP)). Then, the high concentration (20 mg/L) PPCPs solution was diluted to obtain 1 L of an 800 µg/L mixed solution. In adsorption kinetic experiments, 2 mg of the activated MOFs were added to a glass flask with 20 mg/L, 400 µg/L, and 800 µg/L of pollutants in a 50 mL reaction volume. Photodegradation was performed in a sunlight irradiation simulator (AM 1.5) after achieving the adsorption equilibrium in a photoreactor (CEL‐LB50, China). The photocatalysis process was implemented as follows: a Xenon lamp (CEL‐PE300L‐3A) as the light source with a height of 11 cm, 159.0 mW/cm^2^ of optical intensity, and 20 A of current. The reaction solution was stirred at a speed of 250 rpm at room temperature. Finally, the data were collected using high‐performance liquid chromatography (HPLC, Agilent 1260) or HPLC‐mass spectrometry (HPLC‐MS) and fitted to kinetic models.

### 4.6 Statistical Analysis

All experiments were performed at least three times with triplicate samples per condition (n = 3). Data and error bars are presented as mean ± standard deviation (SD) to reflect the variability among independent measurements. Data processing and analysis were performed using Origin 2024 (OriginLab Corporation, Northampton, MA, USA). Two‐tailed t‐tests (unequal variances) and ANOVA with Tukey's test were used, with a significance level of p <0.05. To demonstrate the reproducibility of the optimal performance, three independent batches were provided using the same procedure (Figure S39 and Table S22).

### 4.7 Quality Assurance and Quality Control (QA/QC)

Calibration curves (R^2^>0.99) were obtained for each analyte. The isotope‐labeled internal standards of PPCPs were used for the quantification of target compounds. Recovery tests (85%–115%) and triplicate analyses (RSD <5%) confirmed accuracy and precision (Figure S41 and Table S25). Blanks were routinely included to avoid contamination. Further details about matrix effects, calibration curves, LOD/LOQ, retention time, etc. were described and provided in the Supporting Information (Figures S40 and S41 and Tables S23–S28).

## Author Contributions

**Yuxin Lu**: conceptualization, data curation, formal analysis, investigation, methodology, validation, visualization, Writing – original draft. **Xiang Li**: conceptualization, funding acquisition, supervision, project administration, writing, review & editing. Yuhang, Li, **Zhiyi Sun**, **Fanke Tong**, **Zelin Li**: formal analysis, data curation. **Sheng Wang**: data curation, methodology. **Yi Li** & **Shangkun Pei**: resources, methodology. **Xiyan Xu**: methodology, project administration, writing, review & editing. **Chong‐Chen Wang**: methodology, project administration, writing, review & editing. **Bo Wang**: methodology, project administration, writing, review & editing.

## Conflicts of Interest

The authors declare no conflicts of interest.

## Supporting information




**Supporting File**: advs77224‐sup‐0001‐SuppMat.docx.

## Data Availability

The data that support the findings of this study are available from the corresponding author upon reasonable request.
