# Supplementary material for: Bioinspired Porphyrin‐Based Catalysts From Temporospatial Confined Frameworks for Ultrafast Removal of Sulfaclozine
Source: Adv Sci (Weinh). 2026 Aug 30:e77224. Online ahead of print. doi: 10.1002/advs.77224 (PMC13526422; doi:10.1002/advs.77224)
Supplement: Supplementary file 1 [file ADVS-9999-e77224-s001.docx]

Supporting Information for

**Bioinspired Porphyrin-based Catalysts from Temporospatial Confined Frameworks for Ultrafast Removal of Sulfaclozine**

Yuxin Lu ^a^, Yuhang Li^c^, Zhiyi Sun ^a^, Fanke Tong ^a^, Zelin Li ^a^, Sheng Wang ^a^, Shangkun Pei ^a^, Yi Li ^a^, Xiyan Xu ^a^ *, Chong-Chen Wang ^b^*, Bo Wang ^a^ *, Xiang Li ^a^ *

^a^ School of Chemistry and Chemical Engineering, Beijing Institute of Technology, Beijing 100081, China

^b^ Beijing Key Laboratory of Functional Materials for Building Structure and Environment Remediation, Beijing University of Civil Engineering and Architecture, Beijing 100044, China.

^c^ Eco-environment and Resource Efficiency Research Laboratory, School of Environment and Energy, Peking University Shenzhen Graduate School, Shenzhen, Guangdong 518055, PR China.

*Corresponding author at Chemistry and Chemical Engineering, Beijing Institute of Technology, Beijing, 100081, Beijing University of Civil Engineering and Architecture, China. E-mail address: bowang@bit.edu.cn, xiyanxu@bit.edu.cn, chongchenwang@126.com, [lixiang@bit.edu.cn](mailto:lixiang@bit.edu.cn)

**Materials and Methods**

**Preparation of MOF-808-d.**

0.75 mmol ZrCl_4_ and 0.25 mmol H_3_BTC were mixed in a 50 mL glass flask with the screw gap. Then, 8 mL DMF and 4 mL formic acid were added to the above glass reactor. After 15 minutes of 30 ℃ ultrasonic treatment, transfer the screw gap vial into the oven and heat it to 120 ℃ for 24 hours. After cooling to room temperature, the obtained sample was washed with DMF and acetone each for 2 days to remove impurities. Finally, the MOF-808-d powder was activated in a vacuum oven at 80 ℃ for 10 hours.

**Preparation of MOF-808-0.11TCPP membranes.**

10 mg of MOF-808-0.11TCPP powder was added to an anhydrous ethanol, and a deionized water mixed solution (7:1 V/V), then 100 μL Nafion PFSA polymer solution was dropped into the mixed solution. Polytetrafluoroethylene (PVDF) film (Φ=35 mm) is placed in a vacuum filtration device, washed three times with anhydrous ethanol, and then poured into the above mixed solution to evenly disperse the MOF material on the PVDF film. After washing three times with ethanol, the MOF-808-0.11TCPP/PVDF membrane was obtained by drying in an 80 ℃ vacuum oven for 12 hours.

**EXAFS.**

The X-ray absorption fine structure spectra (XAFS) Zr K-edge were collected at the BL14W1 beamline of Shanghai Synchrotron Radiation Facility (SSRF), while Zr K-edge spectra were collected at the 1W1B beamline of Beijing Synchrotron Radiation Facility (BSRF). The data were collected in fluorescence mode using a Lytle detector, while the corresponding reference samples were collected in transmission mode. The sample was ground and uniformly daubed on the special adhesive tape.

The acquired EXAFS data were processed according to the standard procedures using the ATHENA module implemented in the IFEFFIT software packages. The EXAFS spectra were obtained by subtracting the post-edge background from the overall absorption and then normalizing for the edge-jump step. Subsequently, the χ(k) data were Fourier transformed to real (R) space using a hanging window (dk=1.0 Å^-1^) to separate the EXAFS contributions from different coordination shells.

The following EXAFS equation was used:

$$\chi\left( k \right)= \sum_{j} \frac{N_{j}S_{o}^{2}F_{j}(k)}{kR_{j}^{2}} exp[-2k^{2}\sigma_{j}^{2}]exp[\frac{-2R_{j}}{\lambda\left( k \right)}]sin[2kR_{j}+ \phi_{j}(k)]$$

S_0_^2^ is the amplitude reduction factor, F_j_(k) is the effective curved-wave backscattering amplitude, N_j_ is the number of neighbors in the j_th_ atomic shell, R_j_ is the distance between the X-ray absorbing central atom and the atoms in the j_th_ atomic shell (backscattered), λ is the mean free path in Å, ϕ_j_(k) is the phase shift (including the phase shift for each shell and the total central atom phase shift), σ_j_ is the Debye-Waller parameter of the j_th_ atomic shell (variation of distances around the average R_j_). The functions F_j_(k), λ, and ϕ_j_(k) were calculated with the ab initio code FEFF8.2. The coordination numbers of model samples were fixed as the nominal values. The obtained S02 was fixed in the subsequent fitting. While the internal atomic distances R, Debye-Waller factor σ^2^, and the edge-energy shift ΔE_0_ were allowed to run freely.

**Calculation for TGA.**

The chemical formula of MOF-808 is Zr_6_O_4_(OH)_4_(BTC)_2_(HCOO)_6_, with a molecular weight of 1363.7 g/mol. During the TGA process, the structural formula at the first platform corresponding to 350 ℃ changes to Zr_6_O_9_(BTC)_2_, with a molecular weight of 1105.3 g/mol; Ideally, the platform should be found at 149.5% of the TGA trace (relative to the residual mass of 6(ZrO_2_) at 600 ℃). On the second platform at ⁓ 600 ℃, the MOF framework was completely degraded in an air atmosphere, leaving zirconia 6(ZrO_2_) remaining. Molecular weight = 739.3 g/mol; Normalize to 100% on the TGA trace. Therefore, during the TGA measurement process, skeleton disintegration and organic linker loss occur within the temperature range (400 ℃ ~ 600 ℃). The theoretical weight loss of a perfect crystal structure is = (1105.3 – 739.3)/1105.3 × 100 = 33.11% [This weight loss% is equivalent to the loss of 2BTC connectors per secondary building unit (SBU)] for each MOF, an estimate of the weight loss% from 400 ℃ to 600 ℃ for each MOF and then provide the number of missing connectors for each node.

**Adsorption and Photocatalysis performance with HPLC analysis methods.**

The chemicals in the solution were analyzed using High-Performance Liquid Chromatography (HPLC, Agilent 1260 II) coupled with UV-vis detection. The column was Poros hell 120 SB-C18 4.6 mm × 150 mm, 2.7 μm. The flow rate was set at 0.500 mL/min, the column temperature was 30 ℃, and the test time was 15 min. The wavelength of the UV detectors of SCL was 210 nm. The retention time of SCL was 7 min. The mobile phase uses a 0.1% formic acid and 2 mmol/L ammonium acetate aqueous solution (A) and methanol (B). The percentage of mobile phase Volume_A_/Volume_B_ of SCL was 25%/75%.

The equilibrium adsorption capacity and the initial adsorption rate of materials were calculated by Eq. 1 and Eq. 3, respectively.

Pseudo-second order kinetic model:

$Q_{t}\text{ = }\frac{(C_{0}\text{ - C})V}{m}$	(1)

$\frac{t}{Q_{t}}\text{ = }\frac{1}{k_{2}Q_{e}^{2}}\text{ + }\frac{t}{Q_{e}}\text{ = }\frac{1}{v_{0}}\text{ + }\frac{t}{Q_{e}}$	(2)

Initial adsorption rate, quantified in mg·g^-1^·min^-1^:

$v_{0}\text{ = }\text{k}_{2}Q_{e}^{2}$	(3)

Elovich model:

$Q_{t}\text{ = }\frac{1}{B} \times(\text{ln}\text{ }(\text{A }\times\text{ B }\times\text{ t + 1}))$	(4)

Weber-Morris model:

$Q_{t}\text{ = k }\times\text{ }\text{t}^{\frac{1}{2}}\text{ + C}$	(5)

Where Q_t_ (mg/g) represents the amount of adsorption at a specific time point. Where v_0_ (mg·g^-1^·min^-1^) is the initial adsorption rate. Where Q_e_ (mg/g) represents the equilibrium adsorption capacity, c_0_ (mg/L) denotes the initial concentration of the adsorbate, c (mg/L) stands for the concentration measured at a specific sampling point, V (mL) is the total volume of the experimental system, m (g) indicates the utilized adsorbent mass, and t (min) is the reaction time. Where k_2_ and k are the kinetic constants of the pseudo-second-order model and the Weber-Morris model, respectively. A, B, and C are the constants.

The performance under different water matrices (SCL, 20 mg/L, 40 mL) was evaluated by the simulated solution added inorganic salts, KCl, NaNO_3_, Na_2_SO_4_, NaHCO_3_, and Na_2_CO_3_. The influence of pH was studied by adjusting the solution pH to 3.0, 5.0, 7.0, 9.0, and 11.0 using a 10% HCl solution and 2 mol/L NaOH.

The degradation data collected by HPLC was fitted by a pseudo-first-order kinetic linear fitting analysis (Eq. 6).

$\text{ln}(\frac{C}{C_{0}})\text{ = }k_{app}\text{t + B}$	(6)

Where c represents the concentration of solution at the specific time point, c_0_ means the initial concentration of reaction solution, t is the reaction time, k_app_ represents the kinetic constant of the pseudo-first-order model, and B is the constant.

**Detection conditions of liquid chromatograph-mass spectrometer (HPLC-MS)**

The flow rate of the mobile phase was 0.2 mL/min. The sample was detected in positive or negative electrospray ionization mode after passing through the Eclipse Plus C18 1.8 µm column (2.1 mm × 50 mm). Liquid chromatography-mass spectrometry (LC-MS) is a commonly used method for analyzing trace pollutants. After separation in a chromatographic column, the sample undergoes partial mobile phase separation by mass spectrometry. After ionization, the mass-to-charge to m/z of different ion fragments is obtained by a mass analyzer. At the same time, the concentration of known mass to substances is quantitatively analyzed by the comparative curve method.

The separation process adopts a liquid phase series elution program, and the mobile phase uses a 0.1% formic acid and 2 mmol/L ammonium acetate aqueous solution (A) and acetonitrile (B). The detailed mass spectrometry detection parameters of pollutants are shown in **Table S23**.

**Electrochemical Measurements**

The electrochemical measurements were performed with an electrochemical workstation (CHI-660E, China) in a three-electrode system with Na_2_SO_4_ solution (0.5 M) as the electrolyte, an Ag/AgCl electrode as the reference electrode, a platinum plate electrode as the counter electrode, and TCPP@MOF-808 as a working electrode. The working electrodes were prepared with MOFs (2.0 mg) and Nafion/ethanol solution (volume ratio of 1:50, 200 µL) and treated by sonication for 10 min. Then, 50 µL of the above solution was evenly coated onto FTO conductive glass (1×1 cm^2^) and dried at room temperature.

**Quenching experiment and quantitative probe tests**

Specific quenching agents were employed to quench the different reactive species in the system of MOF-808-0.11TCPP and MOF-808-0.21TCPP and SCL (20 mg/L) during the photocatalytic experiment: potassium bromate (10 mM for electrons (e^-^), superoxide dismutase (SOD, 0.16 g/L) for superoxide radicals (O_2_^•⁻^), ammonium oxalate (20 mM) for hydrogen ions (h^+^), L-tryptophan (10 mM) for singlet oxygen (^1^O_2_), and tert-butanol (TBA, 10 mM) for hydroxyl radicals (•OH). After an adsorption process of 2.5 hours to ensure the adsorption reaction reached equilibrium, the quenchers were added to the solution, and the photocatalytic experiment was carried out for 1 hour. To test the steady-state concentration of reactive species, furfuryl alcohol (FFA, 50 μM), nitrobenzene (NB, 50 μM), and nitro tetrazolium blue chloride (NBT, 50 μM) were used to test the non-free radical (^1^O_2_) and free radicals (•OH and O_2_^•−^) in the system.

**Computational method.**

The density functional theory (DFT) is performed by the Vienna Ab Initio Simulation Package (VASP). The plausible structure of the Zr cluster with TCPP and defects is established from theoretical study. The projected augmented wave (PAW) potentials were chosen to describe the ionic cores. Valence electrons were calculated by a plane wave basis set with 400 eV. The Gaussian smearing method was used for partial occupancies of the Kohn−Sham orbitals (0.05 eV width). When the energy change was less than 10 ~ 5 eV, the electronic energy (EE) was considered self-consistent. W the force change was less than 0.02 eV/Å, a geometry optimization was considered convergent. The dispersion interactions were described by Grimme’s DFT-D3 methodology.

The MOF-808 cluster, which comprises 42 Zr, 82 C, 40 H, and 136 O atoms, was in a tetragonal box of 32 Å × 32 Å × 42 Å. During structural optimizations, the Γ point in the Brillouin zone was used for k-point sampling, and all atoms were allowed to relax.

The adsorption energy (Eads) of adsorbate A was defined as

E_ads_ = E_A/surf_ – E_surf_ – E_A(g)_

where E_A/surf_, E_surf,_ and E_A(g)_ are the energy of adsorbate A adsorbed on the surface, the energy of a clean surface, and the energy of an isolated A molecule in a cubic periodic box with a side length of 20 Å and a 1×1×1 Monkhorst-Pack k-point grid for Brillouin zone sampling, respectively.

**The method of recovery efficiency tests**

The internal standard was obtained by diluting the mixed stock with methanol. For sample pretreatment, water samples were filtered through glass fiber filters (pre-combusted at 450 °C for 4.5 h). An 1000 mL of the filtered water was spiked with 100 μL of the internal standard working solution, mixed thoroughly, and the pH was adjusted using 1 mol/L NaOH or HCl. Solid-phase extraction (SPE) cartridges were sequentially conditioned and equilibrated with 3 × 6 mL of methanol and 3 × 6 mL of ultrapure water at a flow rate of 6 mL/min. The spiked water samples were then loaded onto the cartridges at a flow rate of 3–6 mL/min. After loading, the cartridges were rinsed with 5 mL ultrapure water and vacuum-dried for 1.5–2 h to remove residual water. The target analytes were eluted with 8 mL of methanol containing 0.1% formic acid (optimized solvent and volume) at a flow rate of 0.5 mL/min, and the eluate was collected in a 10 mL glass graduated centrifuge tube with a screw cap. The eluate was concentrated to near dryness under a gentle stream of high-purity nitrogen in a water bath at 38 °C, reconstituted to 1 mL with methanol, vortex-mixed for 1 min, and transferred to an autosampler vial for HPLC-MS/MS analysis. Recovery tests were performed using the same optimized conditions in different water samples.

**QA/QC**

To meet the standards of environmental analytical chemistry, a rigorous QA/QC protocol was established. All experiments were conducted in high-purity water (18.2 MΩ·cm, no additional matrix), making matrix effects negligible. The following validation parameters were evaluated.

(1) Calibration curves and external standard quantification
For each target pollutant, an external standard calibration curve was constructed over the concentration range of 0.25~400 μg/L. Raw data of the standard samples (including concentration (Conc.), Retention time (RT), and accuracy) are provided in **Figure S40 and Table S23**. All calibration curves exhibited coefficients of determination (R^2^) > 0.99.

(2) Limits of detection and quantification
Limits of detection (LOD) and quantification (LOQ) were determined using the signal-to-noise (S/N) ratio method in high-purity water. LOD was defined as the concentration at S/N = 3, and LOQ as S/N = 10. The LOQ was around 3.7E-07~0.195 μg/L. The LOD was around 1.1E-07~0.0585 μg/L.

(3) Recovery rate and Matrix effects
The most reaction system consisted of high-purity water, target pollutants, and photocatalyst (removed by 0.22 μm filtration after reaction). No complex dissolved matrix (e.g., humic substances, inorganic salts) was present in HPLC-MS. The internal standard of PPCPs was used to validate the quantification of targeted compounds, which was shown in **Table S21**. The recovery rate in ultrapure water of PPCPs was shown in **Figure S41**. Recovery rate and LOD/LOQ were tested to evaluate the matrix effects in distilled water, tap water, and lake water were shown in **Table S24-S28**.

(5) Reproducibility and quality control samples
Each batch of sample analysis included: Method blank (high-purity water) and triplicate samples (one replicate inserted every three samples; relative standard deviation < 5%) as shown in **Figure S39 and Table S22**. All quantitative results are reported as mean ± standard deviation (SD) from three independent replicate experiments.

**Figure S1.** Adsorption energy of monodentate coordination and bidentate coordination of TCPP@MOF-808.

**Figure. S2** The digital graphs during the synthesis and washing process. The serial number **0~5** corresponds to MOF-808, MOF-808-0.05TCPP, MOF-808-0.08TCPP, MOF-808-0.11TCPP, MOF-808-0.16TCPP, and MOF-808-0.21TCPP from left to right.

**Figure. S3** Fourier transform infrared spectroscopy (FTIR) of as-prepared MOF-808-TCPP and MOF-808.

**Figure. S4** The XPS full spectrum of MOF-808, MOF-808-0.11TCPP, and MOF-808-0.21TCPP.

**Figure. S5** Pore size distribution calculated by the QSDFT model and the total pore volume.

**Figure. S6** Scanning electron microscopy (SEM) images of (a) MOF-808, (b) MOF-808-0.05TCPP, (c) MOF-808-0.08TCPP, (d) MOF-808-0.11TCPP, (e) MOF-808-0.16TCPP, (f) MOF-808-0.21TCPP.

**Figure. S7** The elemental analysis of MOF-808-TCPP.

\

**Figure. S8** The TGA curves of MOF-808, MOF-808-0.05TCPP, MOF-808-0.08TCPP, MOF-808-0.11TCPP, MOF-808-0.16TCPP, and MOF-808-0.21TCPP.

**Figure. S9** The TGA calculation results of MOF-808-TCPP and MOF-808.

**Figure. S10** The O 1s spectrum of MOF-808, MOF-808-0.11TCPP, and MOF-808-0.21TCPP.

**Figure. S11** EXAFS spectra and the fitting curves of MOF-808 at the (a) *K* space and (b) *R* space.

**Figure. S12** EXAFS spectra and the fitting curves of MOF-808-0.11TCPP at the (a) *K* space and (b) *R* space.

**Figure. S13** The enlarged EXAFS spectra of MOF-808 and MOF-808-0.11TCPP.

**Figure. S14** The 1H-NMR spectrum of (a) MOF-808, (b) MOF-808-0.05TCPP, (c) MOF-808-0.08TCPP, (d) MOF-808-0.11CPP, (e) MOF-808-0.16TCPP, (f) MOF-808-0.21TCPP, digested in 2 M NaOH solution prepared in D_2_O.

**Figure. S15** The coordination number of BTC, TCPP, HCOOH, and compensated ligands of MOF-808-0.05TCPP, MOF-808-0.08TCPP, and MOF-808-0.16TCPP.

**Figure. S16** (**a)** adsorption kinetics of SCL and **(b-d)** Weber-Morris model of SCL adsorption. Reaction conditions: reaction volume of 50 mL, the initial concentration of KP was 800 μg/L, catalyst dosage of 2 mg.

**Figure. S17** (a) adsorption kinetics of KP, (b) the pseudo-second order kinetic model of KP adsorption, (c) the Elovich model of KP adsorption, (d-i) the Weber-Morris model of KP adsorption. Reaction conditions: reaction volume of 50 mL, the initial concentration of KP was 800 μg/L, catalyst dosage of 2 mg.

**Figure. S18 (**a) adsorption kinetics of DF, (b) the pseudo-second order kinetic model of DF adsorption, (c) the Elovich model of DF adsorption, (d-i) the Weber-Morris model of DF adsorption. Reaction conditions: reaction volume of 50 mL, the initial concentration of DF was 800 μg/L, catalyst dosage of 2 mg.

**Figure. S19** (a) adsorption kinetics of IBU, (b) the pseudo-second order kinetic model of IBU adsorption, (c) the Elovich model of IBU adsorption, (d-i) the Weber-Morris model of IBU adsorption. Reaction conditions: reaction volume of 50 mL, the initial concentration of IBU was 800 μg/L, catalyst dosage of 2 mg.

**Figure. S20** (a) adsorption kinetics of CA, (b) the pseudo-second order kinetic model of CA adsorption, (c) the Elovich model of CA adsorption, (d-i) the Weber-Morris model of CA adsorption. Reaction conditions: reaction volume of 50 mL, the initial concentration of CA was 800 μg/L, catalyst dosage of 2 mg.

**Figure. S21** The saturated adsorption capacity (Q_e_) and the initial adsorption rate (v_0_) of MOF-808-TCPP of KP, DF, CA, and IBU.

**Figure. S22** The adsorption and photodegradation process of SMX, SP, CBZ, and SCL.

**Figure. S23** The adsorption of NFC, SCL, CA, KP, and CFC on MOF-808-0.11TCPP at different pH levels. Reaction conditions: reaction volume is 50 mL, the initial concentration was 400 µg/L, catalyst dosage was 2 mg.

**Figure. S24** (a) the XPS full spectrum of MOF-808-0.11TCPP and MOF-808-0.11TCPP-SCL, (b) the Cl 2p spectrum of MOF-808-0.11TCPP-SCL.

**Figure. S25** The N 1s spectrum of MOF-808-0.11TCPP and MOF-808-0.11TCPP-SCL.

**Figure. S26** The C 1s spectrum of MOF-808-0.11TCPP and MOF-808-0.11TCPP-SCL.

**Figure. S27** The linear fitting of SCL photodegradation. Reaction conditions: reaction volume of 50 mL, the initial concentration of SCL was 400 μg/L, catalyst dosage of 2 mg.

**Figure. S28** The adsorption and photodegradation process of SCL. Reaction conditions: the reaction volume is 40 mL, the initial concentration of SCL was 20 mg/L, the catalyst dosage was 2 mg, and the intensity of visible light was 159.0 mW/cm^2^.

**Figure. S29** Correlation between mesopore/micropore volume and degradation rate constant of sulfaclozine.

**Figure S30** The adsorption and degradation process of SCL by MOF-808+TCPP, MOF-808/TCPP, MOF-808-d, and MOF-808-0.11TCPP which the adsorption process had been normalized.

**MOF-808+TCPP** (**Physical mixture of TCPP and MOF-808):** Free TCPP (same amount as in MOF-808-0.11TCPP) was physically mixed with pristine MOF-808, named as MOF-808+TCPP (mix). The mixture showed negligible enhancement in degradation compared to pristine MOF-808, indicating that simple co-existence of TCPP and MOF-808 without confinement does not yield the high activity.

**MOF-808/TCPP (Surface-loaded TCPP material):** TCPP was loaded onto the external surface of MOF-808 by post-synthetic impregnation (DMF, 120 ℃ for 10 h), named as MOF-808/TCPP. The size of TCPP makes it difficult to transport in the micropore of MOF-808. This material exhibited less activity than the confined counterpart (MOF-808-0.11TCPP), suggesting that the confined location plays a role. It also showed less activity than MOF-808-d because the TCPP on the surface might prevent the transfer of ROS and pollutants.

**MOF-808-d (Defect-only MOF-808 without TCPP):** A defect-rich MOF-808 sample (MOF-808-d) was synthesized by modulating formic acid concentration in the original manuscript. Its activity was only slightly higher than pristine MOF-808 but much lower than MOF-808-0.11TCPP, indicating that defects alone cannot explain the observed enhancement.

**Figure. S31** The adsorption and photodegradation of multiple pharmaceuticals, including ofloxacin (OFC), norfloxacin (NFC), ciprofloxacin (CFC), diclofenac (DF), ibuprofen (IBU), ketoprofen (KP), clofibric acid (CA), and sulfaclozine (SCL) by MOF-808-0.21TCPP. Reaction conditions: reaction volume is 50 mL, the initial concentration was 400 µg/L, catalyst dosage was 2 mg, the intensity of visible light was 159.0 mW/cm^2^.

**Figure. S32 (a) The nitrogen adsorption curves and (b) pore size distribution before and after continuous-flow reaction.** (c) The PXRD of MOF-808-0.11TCPP under different pH levels. (d) The PXRD of MOF-808-0.11TCPP before and after continuous-flow reaction. (e) The Zr 3d spectrum of MOF-808-0.11TCPP after regeneration. (f) The O 1s spectrum of MOF-808-0.11TCPP after regeneration

**Figure. S33** The SCL degradation performance with (a) different catalyst dosage, (b) different concentration, (c) different pH levels on MOF-808-0.11TCPP. (d) The kinetics of SCL degradation with 20 mg/L of different coexisting substances.

**Figure. S34** (a) the quenching test of SCL with different trapping agents of MOF-808-0.11TCPP. (b) The quenching test of SCL with different trapping agents of MOF-808-0.21TCPP.

**Figure. S35** The photodegradation of SCL by MOF-808-0.11TCPP with 0.3 g/L O_2._ Reaction conditions: the reaction volume is 40 mL, the initial concentration of SCL was 20 mg/L, the catalyst dosage was 2 mg, and the intensity of visible light was 159.0 mW/cm^2^.

The degradation of SCL was slightly promoted after 0.3 g/L of oxygen was introduced, and the degradation rate constant was enhanced to 0.0177 min^-1^, illustrating that the sources of oxygen atoms in ROS came from dissolved O_2_ in water.

**Figure S36** The quenching experiment of superoxide anions by p-BQ and the TEMP-^1^O_2_ signal with p-BQ.

**Figure. S37** The Mott-schottky curves of MOF-808-0.05TCPP, MOF-808-0.08TCPP, MOF-808-0.16TCPP, and MOF-808-0.21TCPP.

**Figure. S38** The electron transfer mechanism and the ROS generation in TCPP@MOF-808.

**Figure S39** The reproducibility of MOF-808-0.11TCPP in different batch experiments. (a) PXRD, (b) FTIR spectra, (c) N_2_ adsorption and desorption curves, (d) The SCL adsorption and degradation process. Condition: Reaction conditions: reaction volume of 50 mL, the initial concentration of SCL was 800 μg/L, catalyst dosage of 2 mg.

**Figure S40** The fitting results of external standard calibration of PPCPs.

**Figure S41** The recovery rate of PPCPs in ultrapure water.

**Table S1.** The reagent amounts used in the synthesis of MOF-808-TCPP.

| Samples | ZrCl_4_/mg | H_3_BTC/mg | H_2_TCPP/mg | TCPP:BTC(mol) |
| --- | --- | --- | --- | --- |
| MOF-808 | 175 | 52.8 | 0.00 | 0 |
| MOF-808-0.05TCPP | 175 | 52.8 | 10.56 | 0.05 |
| MOF-808-0.08TCPP | 175 | 52.8 | 15.84 | 0.08 |
| MOF-808-0.11TCPP | 175 | 52.8 | 21.12 | 0.11 |
| MOF-808-0.16TCPP | 175 | 52.8 | 31.68 | 0.16 |
| MOF-808-0.21TCPP | 175 | 52.8 | 42.24 | 0.21 |

**Table S2** FTIR vibrational wavenumbers (cm^-1^) of MOF-808-TCPP.

| Chemical bonds | MOF-808 | 0.05TCPP | 0.08TCPP | 0.11TCPP | 0.16TCPP | 0.21TCPP |
| --- | --- | --- | --- | --- | --- | --- |
| ν (Zr-O-Zr) | 451 | 450 | 450 | 450 | 451 | 451 |
| ν (Zr-O) | 653 | 657 | 655 | 657 | 660 | 659 |
| γ (C=C in benzene ring) | 942 | 943 | 942 | 942 | 942 | 941 |
| γ (C=C in benzene ring) | 1109 | 1110 | 1109 | 1109 | 1109 | 1109 |
| ν_s_ (-COO- in BTC) | 1377 | 1377 | 1378 | 1377 | 1377 | 1377 |
| ν_s_ (-COOH)  δ(C-H) | 1444 | 1444 | 1444 | 1445 | 1444 | 1444 |
| ν_as_ (-COOH)  ν (-COO-)  δ (C-H in BTC) | 1606 | 1604 | 1605 | 1604 | 1605 | 1605 |
| ν_as_ (-COO-Zr)  ν (-C=C-)  ν (-COO-) | 1656 | 1659 | 1657 | 1659 | 1660 | 1660 |

**Table S3** Surface areas and pore size distribution of MOF-808 and various MOF-808-TCPP samples.

| Materials | S_BET_  (m^2^·g^-1^) | Average  pore size (nm) | V_total_  (cm^3^·g^-1^) | V_micro_  (cm^3^·g^-1^) | Ratio (V_micro_) | V_meso_  (cm^3^·g^-1^) | Ratio (V_meso_) |
| --- | --- | --- | --- | --- | --- | --- | --- |
| MOF-808 | 1606 | 1.356 | 1.066 | 0.873 | 81.89% | 0.193 | 18.11% |
| 0.05TCPP | 1325 | 1.416 | 1.027 | 0.778 | 75.75% | 0.249 | 24.25% |
| 0.08TCPP | 1312 | 1.416 | 1.010 | 0.754 | 74.65% | 0.256 | 25.35% |
| 0.11TCPP | 1283 | 1.416 | 0.999 | 0.717 | 71.78% | 0.282 | 28.22% |
| 0.16TCPP | 1231 | 1.416 | 0.952 | 0.701 | 73.63% | 0.251 | 25.32% |
| 0.21TCPP | 1054 | 1.416 | 0.787 | 0.573 | 72.81% | 0.214 | 27.19% |

**Table S4** The content of atoms in MOF-808-TCPP tested by XPS.

| Sample | N at.% | Zr at.% | C at.% | O at.% |
| --- | --- | --- | --- | --- |
| MOF-808 | 0 | 5.76 | 56.15 | 38.09 |
| 0.05TCPP | 0.53 | 5.63 | 57.46 | 36.38 |
| 0.08TCPP | 0.69 | 5.44 | 60.31 | 33.56 |
| 0.11TCPP | 0.91 | 5.23 | 59.35 | 34.52 |
| 0.16TCPP | 1.32 | 4.71 | 62.85 | 31.12 |
| 0.21TCPP | 1.67 | 4.82 | 62.33 | 31.18 |

**Table S5:** The weight loss% and the calculated number of BTC and integrating TCPP of various MOF-808-TCPP samples by TGA analysis.

| Sample | Weight loss%(Ⅰ) | Weight loss%(Ⅱ) | Number of BTC | Number of TCPP | Number of HCOOH |
| --- | --- | --- | --- | --- | --- |
| MOF-808 | 39.34 | 47.79 | 1.93 | 0 | 1.86 |
| 0.05TCPP | 45.55 | 54.49 | 1.84 | 0.14 | 1.52 |
| 0.08TCPP | 47.37 | 55.87 | 1.51 | 0.19 | 0.55 |
| 0.11TCPP | 48.08 | 61.30 | 1.44 | 0.26 | 0.34 |
| 0.16TCPP | 50.99 | 67.64 | 1.08 | 0.42 | 0.33 |
| 0.21TCPP | 56.58 | 72.32 | 0.87 | 0.52 | 0.28 |

**Table S6** The EXAFS fitting result of Zr K-edge (S_0_^2^ = 0.87).

| Sample | Path | CN | R(Å) | σ^2^(10^-3^Å^2^) | ΔE_0_(eV) | R factor |
| --- | --- | --- | --- | --- | --- | --- |
| MOF-808 | Zr-O^1st^ | 3.4 | 2.20 | 6.7 | 5.2 | 0.011 |
|  | Zr-Zr/Zr-O^2nd^ | 5.6 | 2.71 | 6.9 | 4.7 |  |
| MOF-808-0.11TCPP | Zr-O^1st^ | 3.7 | 2.18 | 5.2 | 4.1 | 0.013 |
|  | Zr-Zr/Zr-O^2nd^ | 5.9 | 2.69 | 4.4 | 5.2 |  |

S_0_^2^ is the amplitude reduction factor; CN is the coordination number; R is the interatomic distance (the bond length between Zr central atoms and surrounding coordination atoms); σ^2^ is the Debye-Waller factor (a measure of thermal and static disorder in absorber-scatterer distances); ΔE_0_ is edge-energy shift (the difference between the zero kinetic energy value of the sample and that of the theoretical model). R factor is used to evaluate the goodness of the fit.

This value was fixed during EXAFS fitting, based on the known structure. Error bounds that characterize the structural parameters obtained by EXAFS spectroscopy were estimated as N ± 20%, R ± 1%, σ^2^ ± 20%, and ΔE_0_ ± 20%.

**Table S7** The properties and structure of pharmaceuticals.

| PPCPs | Full name | pK_a_ | Structure | Molecular size (Å×Å×Å) |
| --- | --- | --- | --- | --- |
| CA | clofibric acid | 3.2 |  | 7.66×4.21×4.7 |
| KP | ketoprofen | 5.9 |  | 10.32×7.49×4.62 |
| DF | diclofenac | 4.2 |  | 9.74×8.04×5.46 |
| IBU | ibuprofen | 4.5 |  | 9.47×4.41×4.43 |
| IM | Indomethacin | 4.5 |  | 12.8×9.9×4.3 |
| OFC | ofloxacin | 5.8/8.4 |  | 15.2×7.4×4.2 |
| SCL | sulfaclozine | 2.0/5.5 |  | 7.12×5.46×4.23 |
| NFC | norfloxacin | 6.4/8.8 |  | 13.18×7.58×3.84 |
| CFC | ciprofloxacin | 6.1/8.6 |  | 13.18×8.03×3.84 |

**Table S8** Adsorption kinetics of SCL of various MOF-808-TCPP samples fitting by pseudo-second-order model, Elovich model, and Weber-Morris model.

| SCL |  | 0.05TCPP | 0.08TCPP | 0.11TCPP | 0.16TCPP | 0.21TCPP | MOF-808 |
| --- | --- | --- | --- | --- | --- | --- | --- |
| PSO | Q_e_ | 1098.3 | 1248.5 | 1298.9 | 764.8 | 693.3 | 694.4 |
|  | v_0_ | 475.3 | 1466.9 | 2092.3 | 424.6 | 92.7 | 334.6 |
|  | R^2^ | 0.99 | 0.99 | 0.99 | 0.99 | 0.96 | 0.99 |
| Elovich | a | 6721.08 | 3.35E+07 | 5.97E+08 | 85969 | 125.01 | 2941.61 |
|  | b | 0.0074 | 0.0135 | 0.0155 | 0.0149 | 0.0059 | 0.0104 |
|  | R^2^ | 0.99 | 0.93 | 0.91 | 0.96 | 0.99 | 0.96 |
| W-M | ki_1_ | 350.64 | 474.64 | 557.69 | 221.40 | 156.25 | 242.43 |
|  | Ci_1_ | 0.03 | 0.00 | 0.11 | -0.09 | -0.11 | 0.00 |
|  | R^2^ | 0.99 | 0.99 | 0.99 | 0.97 | 0.92 | 0.99 |
|  | ki_2_ | 54.08 | - | - | - | - | 45.45 |
|  | Ci_2_ | 704.63 | - | - | - | - | 342.58 |
|  | R^2^ | 0.79 | - | - | - | - | 0.79 |
|  | Ci_3_ | 1199.16 | 1151.22 | 1240.62 | 661.67 | 611.04 | 687.12 |

**Notes:** PSO represents the pseudo-second-order model. W-M represents the Weber-Morris model. Q_e_ (μg/g) means equilibrium adsorption capacity, v_0_ (μg/g·min) represents initial adsorption rate. ki_1_, ki_2_, and ki_3_ (mg·g^-1^·min^-0.5^) are the kinetic constants of different phases of the Weber-Morris model.

**Table S9** Adsorption kinetics of KP of MOF-808-TCPP fitting by pseudo-second-order model, Elovich model, and Weber-Morris model.

| KP |  | 0.05TCPP | 0.08TCPP | 0.11TCPP | 0.16TCPP | 0.21TCPP | MOF-808 |
| --- | --- | --- | --- | --- | --- | --- | --- |
| PSO | Q_e_ | 19.51 | 21.05 | 20.66 | 17.92 | 18.33 | 24.91 |
|  | v_0_ | 7.99 | 11.35 | 11.99 | 2.68 | 1.68 | 1.43 |
|  | R^2^ | 0.96 | 0.99 | 0.99 | 0.99 | 0.98 | 0.99 |
| Elovich | a | 140.44 | 11154.18 | 1043.20 | 4.46 | 3.93 | 1.77 |
|  | b | 0.44 | 0.64 | 0.52 | 0.27 | 0.28 | 0.14 |
|  | R^2^ | 0.96 | 0.96 | 0.95 | 0.99 | 0.98 | 0.98 |
| W-M | ki_1_ | 7.61 | 7.04 | 7.11 | 3.60 | 2.34 | 3.23 |
|  | Ci_1_ | -0.02 | -0.03 | -0.01 | -0.42 | -0.29 | -1.71 |
|  | R^2^ | 0.99 | 0.97 | 0.99 | 0.98 | 0.98 | 0.97 |
|  | ki_2_ | 0.94 | 1.92 | 2.40 | 1.62 | 1.67 | 0.62 |
|  | Ci_2_ | 14.48 | 6.99 | 11.28 | 4.10 | 3.48 | 14.46 |
|  | R^2^ | 0.96 | 0.98 | 0.91 | 0.99 | 0.99 | 0.86 |
|  | ki_3_ | 0.005 | 0.05 | -0.04 | -0.03 | 0.12 | —— |
|  | Ci_3_ | 21.03 | 18.40 | 20.21 | 17.87 | 15.20 | —— |

**Notes:** PSO represents the pseudo-second-order model. W-M represents the Weber-Morris model. Q_e_ (mg/g) means equilibrium adsorption capacity, v_0_ (mg/g·min) represents initial adsorption rate. ki_1_, ki_2_, and ki_3_ (mg·g^-1^·min^-0.5^) are the kinetic constants of different phases of the Weber-Morris model.

**Table S10** Adsorption kinetics of DF of MOF-808-TCPP fitting by pseudo-second-order model, Elovich model, and Weber-Morris model.

| DF |  | 0.05TCPP | 0.08TCPP | 0.11TCPP | 0.16TCPP | 0.21TCPP | MOF-808 |
| --- | --- | --- | --- | --- | --- | --- | --- |
| PSO | Q_e_ | 18.62 | 20.64 | 19.57 | 17.94 | 19.32 | 22.13 |
|  | v_0_ | 4.62 | 7.05 | 9.38 | 1.73 | 2.02 | 1.34 |
|  | R^2^ | 0.99 | 0.99 | 0.98 | 0.95 | 0.96 | 0.94 |
| Elovich | a | 22.36 | 73.72 | 322.58 | 3.51 | 3.04 | 2.10 |
|  | b | 0.33 | 0.36 | 0.48 | 0.25 | 0.22 | 0.20 |
|  | R^2^ | 0.97 | 0.94 | 0.96 | 0.97 | 0.99 | 0.98 |
| W-M | ki_1_ | 5.86 | 5.73 | 6.40 | 2.71 | 2.66 | 2.39 |
|  | Ci_1_ | 0.41 | -0.50 | 0.49 | 1.03 | 0.69 | 0.29 |
|  | R^2^ | 0.99 | 0.99 | 0.99 | 0.95 | 0.99 | 0.95 |
|  | ki_2_ | 2.28 | 2.55 | 2.94 | 1.82 | 1.92 | 0.33 |
|  | Ci_2_ | 7.70 | 3.81 | 8.71 | 3.13 | 3.04 | 15.49 |
|  | R^2^ | 0.97 | 0.99 | 0.97 | 0.99 | 0.98 | 0.78 |
|  | ki_3_ | 0.04 | 0.29 | 0.01 | 0.02 | 0.38 | —— |
|  | Ci_3_ | 19.00 | 15.20 | 18.55 | 17.74 | 13.78 | —— |

**Notes:** PSO represents the pseudo-second-order model. W-M represents the Weber-Morris model. Q_e_ (mg/g) means equilibrium adsorption capacity, v_0_ (mg/g·min) represents initial adsorption rate. ki_1_, ki_2_, and ki_3_ (mg·g^-1^·min^-0.5^) are the kinetic constants of different phases of the Weber-Morris model.

**Table S11** Adsorption kinetics of IBU of MOF-808-TCPP fitting by pseudo-second-order model, Elovich model, and Weber-Morris model.

| IBU |  | 0.05TCPP | 0.08TCPP | 0.11TCPP | 0.16TCPP | 0.21TCPP | MOF-808 |
| --- | --- | --- | --- | --- | --- | --- | --- |
| PSO | Q_e_ | 19.44 | 21.98 | 20.36 | 17.8 | 16.21 | 20.61 |
|  | v_0_ | 6.17 | 14.5 | 23.52 | 3.8 | 2.52 | 1.58 |
|  | R^2^ | 6.17 | 14.5 | 23.52 | 3.8 | 2.52 | 1.58 |
| Elovich | a | 47.64 | 4449.23 | 1.84E+14 | 32.37 | 7.75 | 3.86 |
|  | b | 0.36 | 0.59 | 1.89 | 0.42 | 0.34 | 0.25 |
|  | R^2^ | 0.94 | 0.73 | 0.92 | 0.91 | 0.99 | 0.94 |
| W-M | ki_1_ | 8.25 | 6.56 | 7.064 | 4.26 | 2.61 | 2.91 |
|  | Ci_1_ | -1.14 | -0.46 | -0.01 | -1.15 | 1.27 | -0.86 |
|  | R^2^ | 0.99 | 0.99 | 0.99 | 0.96 | 0.98 | 0.99 |
|  | ki_2_ | 2.07 | 2.20 | 0.03 | 1.65 | 0.73 | 0.32 |
|  | Ci_2_ | 10.61 | 6.95 | 19.80 | 7.34 | 9.08 | 14.77 |
|  | R^2^ | 0.96 | 0.99 | —— | 0.99 | 0.99 | 0.65 |
|  | ki_3_ | 0.004 | 0.124 | —— | -0.026 | —— | —— |
|  | Ci_3_ | 19.898 | 17.351 | —— | 17.051 | —— | —— |

**Notes:** PSO represents the pseudo-second-order model. W-M represents the Weber-Morris model. Q_e_ (mg/g) means equilibrium adsorption capacity, v_0_ (mg/g·min) represents initial adsorption rate. ki_1_, ki_2_, and ki_3_ (mg·g^-1^·min^-0.5^) are the kinetic constants of different phases of the Weber-Morris model.

**Table S12** Adsorption kinetics of CA of MOF-808-TCPP fitting by pseudo-second-order model, Elovich model, and Weber-Morris model.

| CA |  | 0.05TCPP | 0.08TCPP | 0.11TCPP | 0.16TCPP | 0.21TCPP | MOF-808 |
| --- | --- | --- | --- | --- | --- | --- | --- |
| PSO | Q_e_ | 15.37 | 16.82 | 16.38 | 15.24 | 13.00 | 17.87 |
|  | v_0_ | 4.04 | 5.75 | 6.91 | 0.83 | 0.75 | 0.76 |
|  | R^2^ | 0.99 | 0.98 | 0.98 | 0.9 | 0.99 | 0.98 |
| Elovich | a | 2.63E+06 | 138.53 | 354.81 | 1.48 | 1.43 | 0.92 |
|  | b | 1.2 | 0.6 | 0.6 | 0.28 | 0.34 | 0.19 |
|  | R^2^ | 0.99 | 0.93 | 0.91 | 0.96 | 0.99 | 0.96 |
| W-M | ki_1_ | 4.96 | 4.56 | 4.77 | 2.11 | 1.58 | 2.19 |
|  | Ci_1_ | -0.54 | -0.32 | -0.15 | -0.32 | -0.23 | -1.17 |
|  | R^2^ | 0.98 | 0.98 | 0.99 | 0.99 | 0.99 | 0.96 |
|  | ki_2_ | 1.52 | 1.71 | 0.11 | 2.46 | 1.33 | 0.58 |
|  | Ci_2_ | 7.76 | 5.60 | 14.73 | 1.15 | 0.97 | 8.14 |
|  | R^2^ | 0.97 | 0.99 | —— | 0.97 | 0.98 | 0.90 |
|  | ki_3_ | 0.001 | 0.21 | —— | —— | -0.07 | —— |
|  | Ci_3_ | 16.05 | 12.68 | —— | —— | 11.71 | —— |

**Notes:** PSO represents the pseudo-second-order model. W-M represents the Weber-Morris model. Q_e_ (mg/g) means equilibrium adsorption capacity, v_0_ (mg/g·min) represents initial adsorption rate. ki_1_, ki_2_, and ki_3_ (mg·g^-1^·min^-0.5^) are the kinetic constants of different phases of the Weber-Morris model.

**Table S13** Degradation kinetics and removal rate of SCL of various MOF-808-TCPP samples by linear fitting.

| SCL Degradation  (400 μg·L^-1^ level) | 0.05TCPP | 0.08TCPP | 0.11TCPP | 0.16TCPP | 0.21TCPP |
| --- | --- | --- | --- | --- | --- |
| k (min ^-1^) | 0.0912 | 0.139 | 0.321 | 0.0681 | 0.0547 |
| removal rate | 100% | 100% | 100% | 100% | 100% |

**Table S14** Degradation kinetics and removal rate of SCL of various MOF-808-TCPP samples by linear fitting.

| SCL Degradation  (20 mg·L^-1^ level) | 0.05TCPP | 0.08TCPP | 0.11TCPP | 0.16TCPP | 0.21TCPP | MOF-808 |
| --- | --- | --- | --- | --- | --- | --- |
| k (min ^-1^) | 0.0123 | 0.0136 | 0.0174 | 0.0105 | 0.0096 | 0.0001 |
| removal rate | 71.5% | 77.0% | 91.3% | 61.4% | 55.7% | 12.3% |
| R^2^ | 0.95 | 0.92 | 0.94 | 0.96 | 0.99 | 0.58 |
| AQE | 7.99% | 8.84% | 11.31% | 6.82% | 6.24% | 0.58% |

**Table S15** The performance of SCL photodegradation of different absorbents.

| Photocatalysts | Condition | Removal rate | Degradation time | Refs. |
| --- | --- | --- | --- | --- |
| 2.0 g/L PDMAA–TiO_2_/CuS | 50 mL 25 mg/L Sulfaclozine | 93.51 % | 16 h | [1] |
| 2.5 g/L TiO_2_ | 25 mL 25 mg/L Sulfaclozine | 94 % | 60 min | [2] |
| 2.5 g/L UV/TiO_2_/K_2_S_2_O_8_ | 25 mL 88 μmol/L Sulfaclozine | 100 % | 40 min | [3] |
| 0.2 g/L Fe-UiO-66/PS | 100 mL 10 mg/L Sulfameter | 88.79 % | 300 min | [4] |
| 1.0 g/L Ag-P@g-C_3_N_4_ | 100 mL 5 mg/L Sulfamethoxazole | 99 % | 30 min | [5] |
| 1.5 g/L N-ZnO/C@BiM  S-scheme heterojunction | 2.5 mg/L Sulfamethoxazole | 96 % | 120 min | [6] |
| 0.1 g/L VNU-1 | 100 mL 800 μg/L Sulfamethoxazole | 100 % | 10 min | [7] |
| 0.05 g/L MOF-808-0.11TCPP | 50 mL 800 μg/L Sulfaclozine | 100 % | 10 min | This work |

**Table S16** The photodegradation rate of micropollutants by the materials reported to date.

| Catalysts | dosage | Targets | Degradation rate | k_app_ | TOF | Ref. |
| --- | --- | --- | --- | --- | --- | --- |
| **MOF-808-0.11TCPP** | **0.04 g/L** | **SCL**  **20 mg/L** | **>95%**  **110 min** | **0.0346 min^-1^** | **1.38×10^-2^ min^-1^** | **This work** |
| TC4-TB10 | 0.08 g/L | STZ  17.8 mg/L | 93.09%  120 min | 0.0805 min^-1^ | 1.79×10^-2^ min^-1^ | [8] |
| Cu-TCPP(BA)-MOF/PMS | 0.1 g/L | TC  20 mg/L | ~85%  40 min | 0.0911 min^-1^ | 1.82×10^-2^ min^-1^ | [9] |
| Cu-TCPP/ ZnFe_2_O_4_ | 1.0 g/L | PNP  50 mg/L | 92.3%  60 min | 0.0812 min^-1^ | 4.06×10^-3^ min^-1^ | [10] |
| 3DNDITcpp | 0.1 g/L | STZ  10 mg/L | ~80%  30 min | 0.054 min^-1^ | 5.4×10^-3^ min^-1^ | [11] |
| PCN-134(Fe) | 0.2 g/L | NPX  40 mg/L | >99%  120 min | 0.049 min^-1^ | 9.8×10^-3^ min^-1^ | [12] |
| CdS@UiO-66(Ce) | 0.08 g/L | DCF  20 mg/L | ~90%  120 min | 0.0288 min^-1^ | 7.20×10^-3^ min^-1^ | [13] |
| 2N-CDs@PCN-222@PNIPAM2 | 0.5 g/L | TC  20 mg/L | ~99%  60 min | 0.0713 min^-1^ | 2.85×10^-3^ min^-1^ | [14] |
| Fe-TCPP/H_2_O_2_ | 0.06 g/L | BPA  10 mg/L | ~90%  60 min | 0.23  min^-1^ | 3.83×10^-2^ min^-1^ | [15] |
| TCPP@UiO-66 | 0.1 g/L | DF  30 mg/L | ~99%  240 min | 0.0084 min^-1^ | 2.52×10^-3^ min^-1^ | [16] |
| PCN-223 | 0.2 g/L | BPF  15 mg/L | ~99%  120 min | 0.0563 min^-1^ | 4.22×10^-3^ min^-1^ | [17] |

**Table S17** The leaching of Zr tested by ICP.

| Sample | The concentration of Zr (μg/L) |
| --- | --- |
| MOF-808-0.11TCPP | 29.198 |
| MOF-808 | 4.666 |

**Table S18** The k_obs_ of SCL degradation under different conditions by MOF-808-0.11TCPP.

| Factors | | Conditions | k_app_ (min^-1^) | R^2^ |
| --- | --- | --- | --- | --- |
| Co-existing ions | | Control | 0.553 | 0.99 |
|  |  | 20 mg/L Cl^-^ | 0.660 | 0.94 |
|  |  | 20 mg/L SO_4_^2-^ | 0.186 | 0.94 |
|  |  | 20 mg/L NO_3_^-^ | 0.225 | 0.95 |
|  |  | 20 mg/L HCO_3_^-^ | 0.025 | 0.98 |
|  |  | 20 mg/L CO_3_^2-^ | 0.051 | 0.96 |
| Actual water | | Control | 0.150 | 0.99 |
|  |  | Distilled water | 0.152 | 0.99 |
|  |  | Tap water | 0.060 | 0.94 |
|  |  | Lake water | 0.053 | 0.94 |
| Initial Conc. | 20 mg/L | 0.05 g/L catalyst  pH 6.8 | 0.016 | 0.98 |
|  | 800 μg/L |  | 0.076 | 0.98 |
|  | 400 μg/L |  | 0.566 | 0.99 |
| MOFs Dosage | 0.20 g/L | 20 mg/L SCL  pH 6.8 | 0.393 | 0.84 |
|  | 0.10 g/L |  | 0.054 | 0.99 |
|  | 0.05 g/L |  | 0.017 | 0.94 |
| pH | 3 | 400 ppb SCL  0.05 g/L catalyst | 0.019 | 0.96 |
|  | 5 |  | 0.462 | 0.99 |
|  | 7 |  | 0.655 | 0.94 |
|  | 11 |  | 0.056 | 0.99 |

**Table S19** The degradation kinetics by quenching and probe tests of MOF-808-0.11TCPP.

| Degradation of SCL | Control | ^1^O_2_(SCL) | ^1^O_2_(FFA) | O_2_^•-^ | •OH | h^+^ | e^-^ |
| --- | --- | --- | --- | --- | --- | --- | --- |
| k_1_ (min^-1^) | 0.0432 | 0.0548 | 0.0121 | 0.0160 | 0.0123 | 0.052 | 0.0284 |
| k_2_ (min^-1^) | 0.0142 | 5.45E-4 | 0.0042 | 0.0103 | / | / | 0.0360 |

**Table S20** The valence band, conduction band, and band gap width of MOF-808-TCPP.

| Materials | MOF-808 | 0.05TCPP | 0.08TCPP | 0.11TCPP | 0.16TCPP | 0.21TCPP |
| --- | --- | --- | --- | --- | --- | --- |
| E_CB_ (V vs. NHE) | -0.41 | -0.47 | -0.43 | -0.42 | -0.40 | -0.37 |
| E_VB_ (V vs. NHE) | 3.42 | 2.33 | 2.35 | 2.35 | 2.34 | 2.32 |
| E_g_ (V vs. NHE) | 3.83 | 2.80 | 2.78 | 2.77 | 2.74 | 2.69 |

**Table S21** The detailed mass spectrometry detection parameters of pollutants.

| Pollutants | Ionization mode | m/z | Crack voltage (V) | Products Ⅰ (m/z) | Products Ⅱ(m/z) | CE Ⅰ (V) | CE Ⅱ (V) | Internal standard |
| --- | --- | --- | --- | --- | --- | --- | --- | --- |
| NFC | ESI+ | 320.1 | 135 | 302.1 | 276.2 | 16 | 21 | C13-pht |
| OFC | ESI+ | 362.2 | 145 | 318.2 | 261.1 | 19 | 29 | C13-pht |
| CFC | ESI+ | 332.1 | 130 | 186.0 | 124.0 | 16 | 27 | C13-pht |
| SCL | ESI+ | 285.0 | 101 | 156.0 | 91.9 | 13 | 33 | C13-pht |
| CA | ESI- | 213.0 | 74 | 127.0 | 84.6 | 16 | 8 | MP-D3 |
| KP | ESI- | 253.1 | 61 | 209.1 | 112.8 | 4 | 36 | MP-D3 |
| DF | ESI- | 294.0 | 75 | 249.9 | 214.2 | 7 | 20 | MP-D3 |
| IM | ESI- | 356.1 | 80 | 312.1 | 297.0 | 5 | 17 | MP-D3 |
| IBU | ESI- | 205.1 | 67 | 161.0 | 154.9 | 0 | 0 | MP-D3 |

**Table S22** The surface area and degradation rate of SCL (C_0_ = 800 μg/L) from different batches.

| Batch | BET surface area (m^2^/g) | Degradation rate of SCL% |
| --- | --- | --- |
| Batch 1 | 1283 | 93.15 |
| Batch 2 | 1207 | 92.10 |
| Batch 3 | 1280 | 91.93 |
| Mean ± SD | 1256±43 | 92.39 ± 0.66 |

The relative standard deviation (RSD) for the degradation rate is < 1.5%, confirming excellent batch-to-batch reproducibility.

**Table S23** The detection results of calibration of PPCPs.

| Data File | RT | NFC Final Conc. | Accuracy% | RT | CA Final Conc. | Accuracy% | RT | OFC Final Conc. | Accuracy% |
| --- | --- | --- | --- | --- | --- | --- | --- | --- | --- |
| 400ppb.d | 4.464 | 391.4496 | 97.9 | 11.098 | 397.0471 | 99.3 | 4.519 | 372.6369 | 93.2 |
| 200ppb.d | 4.485 | 204.5017 | 102.3 | 11.091 | 206.488 | 103.2 | 4.505 | 215.4108 | 107.7 |
| 100ppb.d | 4.499 | 133.655 | 133.7 | 11.084 | 99.8439 | 99.8 | 4.519 | 106.822 | 106.8 |
| 50ppb.d | 4.499 | 45.7792 | 91.6 | 11.091 | 49.3459 | 98.7 | 4.526 | 52.1054 | 104.2 |
| 25ppb.d | 4.506 | 20.1456 | 90.1 | 11.084 | 22.2751 | 93.1 | 4.526 | 25.4637 | 104.9 |
| 12.5ppb.d | 4.495 | 10.8992 | 87.2 | 11.085 | 11.124 | 89.0 | 4.525 | 13.3085 | 122.1 |
| 3.13ppb.d | 4.49 | 3.0127 | 96.3 | 11.085 | 4.2351 | 135.3 | 4.525 | 3.5271 | 117.1 |
| 0.78ppb.d | 4.491 | 0.9354 | 119.9 | 11.084 | 1.3602 | 174.4 | 4.525 | 0.2758 | 29.5 |
| 0.20ppb.d | 4.482 | 0.2153 | 107.7 | 11.091 | 0.2028 | 101.4 | 4.526 | 0.0162 | 8.1 |
| Data File | RT | CFC Final Conc. | Accuracy% | RT | DF Final Conc. | Accuracy% | RT | KP Final Conc. | Accuracy% |
| 400ppb.d | 4.729 | 401.8315 | 100.5 | 12.61 | 390.5471 | 97.6 | 11.272 | 384.8685 | 96.2 |
| 200ppb.d | 4.756 | 175.325 | 87.7 | 12.603 | 219.434 | 109.7 | 11.271 | 231.4045 | 115.7 |
| 100ppb.d | 4.736 | 118.3057 | 118.3 | 12.603 | 103.2071 | 103.2 | 11.264 | 103.9136 | 103.9 |
| 50ppb.d | 4.728 | 50.3352 | 100.7 | 12.603 | 46.1346 | 92.3 | 11.265 | 45.3997 | 90.8 |
| 25ppb.d | 4.729 | 21.5463 | 96.8 | 12.609 | 15.6772 | 62.7 | 11.264 | 29.4136 | 37.7 |
| 12.5ppb.d | 4.73 | 14.0092 | 112.1 | 12.607 | 11.0755 | 88.6 | 11.263 | 17.0287 | 121.6 |
| 3.13ppb.d | 4.727 | 4.3821 | 140.0 | 12.606 | 2.0801 | 66.5 | 11.264 | 3.2113 | 73.3 |
| 0.78ppb.d | 4.721 | 0.7765 | 99.6 | 12.605 | 0.679 | 87.1 | 11.264 | 0.7899 | 101.7 |
| 0.20ppb.d | 4.727 | 0.0234 | 11.7 | 12.606 | 0.136 | 68.0 | 11.265 | 0.298 | 149.0 |
| Data File | RT | SCL Final Conc. | Accuracy% | RT | IM Final Conc. | Accuracy | RT | IBU Final Conc. | Accuracy% |
| 400ppb.d | 7.713 | 389.0462 | 97.3 | 12.624 | 375.1658 | 93.8 | 12.771 | 388.8827 | 97.2 |
| 200ppb.d | 7.706 | 222.5116 | 111.3 | 12.623 | 205.8242 | 102.9 | 12.764 | 223.913 | 112.0 |
| 100ppb.d | 7.706 | 104.1768 | 104.2 | 12.625 | 97.7532 | 97.8 | 12.771 | 100.8994 | 100.9 |
| 50ppb.d | 7.699 | 44.1962 | 88.4 | 12.623 | 38.4835 | 77.0 | 12.764 | 46.6695 | 93.3 |
| 25ppb.d | 7.699 | 15.0693 | 60.3 | 12.623 | 17.7733 | 117.9 | 12.771 | 14.6353 | 58.5 |
| 12.5ppb.d | 7.7 | 12.9815 | 103.9 | 12.62 | 12.9456 | 99.7 | 12.77 | 10.2735 | 79.4 |
| 3.13ppb.d | 7.7 | 4.2596 | 136.1 | 12.619 | 3.0926 | 72.6 | 12.765 | 5.3978 | 174.5 |
| 0.78ppb.d | 7.701 | 0.662 | 84.9 | 12.618 | 1.5714 | 237.4 | 12.763 | 2.3976 | 152.6 |
| 0.20ppb.d | 7.702 | 0.0531 | 26.6 | 12.61 | 0.1952 | 97.6 | 12.765 | 0.1902 | 97.4 |

**Table S24** The quantitation and detection limits of PPCPs in pure water.

| Pharmaceuticals | Abbr. | Limits of Quantitation (μg/L) | Limits of Detection (μg/L) |
| --- | --- | --- | --- |
| Clofibric acid | CA | 0.097 | 0.0291 |
| Diclofenac | DF | 0.012 | 0.0036 |
| Ibuprofen | IBU | 0.19 | 0.057 |
| Ketoprofen | KP | 3.7E-07 | 1.1E-07 |
| Indomethacin | IM | 0.195 | 0.0585 |
| Norfloxacin | NFC | 0.0488 | 0.01464 |
| Ciprofloxacin | CFC | 0.0244 | 0.00732 |
| Ofloxacin | OFC | 6E-05 | 1.8E-05 |
| Sulfaclozine | SCL | 0.0977 | 0.02931 |

**Table S25** The recovery rate (RR) of PPCPs in pure water.

| PPCP | RR% I | RR% II | RR% III | RR% average | RSD % |
| --- | --- | --- | --- | --- | --- |
| NFC | 105.0 | 109.0 | 107.0 | 107.0 | 1.9 |
| OFC | 98.0 | 89.0 | 93.5 | 93.5 | 4.8 |
| CFC | 98.0 | 99.0 | 98.5 | 98.5 | 0.5 |
| SCL | 99.6 | 118.1 | 108.9 | 108.9 | 8.5 |
| IBU | 101.0 | 115.0 | 114.0 | 110.0 | 7.1 |
| KP | 90.0 | 100.8 | 101.4 | 97.4 | 6.6 |
| DF | 89.0 | 95.0 | 97.0 | 93.7 | 4.4 |
| IM | 85.0 | 80.0 | 83.0 | 82.7 | 3.0 |
| CA | 105.0 | 93.0 | 100.0 | 99.3 | 6.1 |

**Table S26** The content of ions, TOC, and COD in different water samples.

| Parameter | Distilled Water (mg/L) | Tap Water (mg/L) | Lake Water (mg/L) |
| --- | --- | --- | --- |
| Na⁺ | < 0.1 | 5.1 | 69.6 |
| K⁺ | < 0.1 | 1 | 7 |
| Ca^2^⁺ | < 0.1 | 17.8 | 73.4 |
| Mg^2^⁺ | < 0.1 | 10 | 22 |
| NH_4_⁺ | < 0.1 | N/A | 0.3 |
| Cl⁻ | < 0.1 | 5.9 | 90.6 |
| SO_4_^2^⁻ | < 0.1 | 45 | 126.2 |
| HCO_3_⁻ | < 0.1 | N/A | 213.5 |
| NO_3_⁻ | < 0.1 | 3.8 | 3.4 |
| F⁻ | 0.02 | 0.3 | 0.4 |
| PO_4_^3^⁻ | < 0.01 | < 0.1 | 0.7 |
| TOC | < 0.5 | 2 | 6.4 |
| COD | < 5 | 11 | 116.3 |

**Table S27** The recovery rate (RR) of PPCPs in different water samples.

| PPCP | Range  (μg/L) | R^2^ | Distilled water | | Tap water | | Lake water | |
| --- | --- | --- | --- | --- | --- | --- | --- | --- |
|  |  |  | Relative RR% | RSD% | Relative RR% | RSD % | Relative RR% | RSD % |
| NFC | 2-400 | 0.9998 | 111.5 | 1.23 | 114.37 | 3.33 | 88.03 | 1.27 |
| OFC | 2-400 | 0.9997 | 94.8 | 3.03 | 95.59 | 1.41 | 78.27 | 2.14 |
| CFC | 2-400 | 0.9959 | 97.66 | 3.53 | 90.8 | 2.81 | 76.24 | 3.02 |
| SCL | 2-400 | 0.9999 | 106.66 | 0.01 | 94.24 | 1.14 | 85.39 | 1.81 |
| IBU | 2-400 | 0.9998 | 114.29 | 7.89 | 94.01 | 0.83 | 83.98 | 0.81 |
| KP | 2-400 | 0.9991 | 96.52 | 2.96 | 100.01 | 3.97 | 76.49 | 3.92 |
| CA | 2-400 | 0.9992 | 94.42 | 1.73 | 99.15 | 0.54 | 82.85 | 2.31 |
| DF | 2-400 | 0.9987 | 98.52 | 2.12 | 96.48 | 1.69 | 75.41 | 0.64 |
| IM | 2-400 | 0.9991 | 98.53 | 1.03 | 100 | 5.26 | 91.1 | 6.95 |

**Table S28** The LOQ and LOD of PPCPs in different water samples.

| PPCPs | Distilled water | | Tap water | | Lake water | |
| --- | --- | --- | --- | --- | --- | --- |
|  | LOQ (μg/L) | LOD (μg/L) | LOQ (μg/L) | LOD (μg/L) | LOQ (μg/L) | LOD (μg/L) |
| NFC | 0.23 | 0.11 | 0.30 | 0.15 | 0.84 | 0.42 |
| OFC | 0.11 | 0.02 | 0.12 | 0.02 | 0.36 | 0.07 |
| CFC | 0.26 | 0.13 | 0.27 | 0.14 | 1.01 | 0.51 |
| SCL | 0.03 | 0.03 | 0.03 | 0.03 | 0.04 | 0.04 |
| IBU | 0.12 | 0.02 | 0.16 | 0.03 | 0.15 | 0.03 |
| KP | 1.42 | 0.28 | 1.47 | 0.29 | 1.34 | 0.27 |
| CA | 1.32 | 0.26 | 1.35 | 0.27 | 1.39 | 0.28 |
| DF | 1.27 | 0.25 | 1.52 | 0.30 | 1.71 | 0.34 |
| IM | 1.16 | 0.23 | 1.47 | 0.29 | 1.42 | 0.28 |

**Reference**

1. X. Guo, F. Yang, X. Sun, C. Han, Y. Bai, G. Liu, W. Liu, R. Wang, "Fabrication of a novel separation-free heterostructured photocatalyst with enhanced visible light activity in photocatalytic degradation of antibiotics", *Journal of Materials Chemistry A*, 10 (2022): 3146-3158. http://dx.doi.org/10.1039/d1ta09757a.

2. L. Ismail, A. Rifai, C. Ferronato, L. Fine, F. Jaber, J.-M. Chovelon, “Towards a better understanding of the reactive species involved in the photocatalytic degradation of sulfaclozine”, *Appl. Catal. B-Environ.* 185 (2016): 88-99. http://dx.doi.org/10.1016/j.apcatb.2015.12.008.

3. L. Ismail, C. Ferronato, L. Fine, F. Jaber, J.-M. Chovelon, “Elimination of sulfaclozine from water with SO^4−^ radicals: Evaluation of different persulfate activation methods”, *Applied Catalysis B: Environmental*, 201 (2017): 573-581. http://dx.doi.org/10.1016/j.apcatb.2016.08.046.

4. Y. Lin, Y. Zhang, G. Li, “Promotion of sulfameter degradation by coupling persulfate and photocatalytic advanced oxidation processes with Fe-doped MOFs”, *Separation and Purification Technology*, 282 (2022): 119632. http://dx.doi.org/10.1016/j.seppur.2021.119632.

5. T.-B. Nguyen, C.P. Huang, R.-a. Doong, C.-W. Chen, C.-D. Dong, “Visible-light photodegradation of sulfamethoxazole (SMX) over Ag-P-codoped g-C_3_N_4_ (Ag-P@UCN) photocatalyst in water”, *Chemical Engineering Journal*, 384 (2020): 123383. http://dx.doi.org/10.1016/j.cej.2019.123383.

6. A. Wang, J. Ni, W. Wang, X. Wang, D. Liu, Q. Zhu, “MOF-derived N-doped ZnO carbon skeleton@hierarchical Bi2MoO6 S-scheme heterojunction for photodegradation of SMX: Mechanism, pathways and DFT calculation”, *Journal of Hazardous Materials*, 426 (2022): 128106. http://dx.doi.org/10.1016/j.jhazmat.2021.128106.

7. J.-X. Tan, Z.-Y. Chen, C.H. Chen, M.-F. Hsieh, A.Y.-C. Lin, S.S. Chen, K.C.W. Wu, “Efficient adsorption and photocatalytic degradation of water emerging contaminants through nanoarchitectonics of pore sizes and optical properties of zirconium-based MOFs”, *Journal of Hazardous Materials*, 451 (2023): 131113. http://dx.doi.org/10.1016/j.jhazmat.2023.131113.

8. Yang, P., Wu, C., Zhang, X., et al., "Enhanced photocatalytic degradation of sulfathiazole via dual-ligand Zr-MOFs: A porphyrin and pyrene-based energy transfer," *Separation and Purification Technology* 354 (2025): 128727. http://dx.doi.org/10.1016/j.seppur.2024.128727.

9. Bai, Y., Nie, G., He, Y., et al., "Cu-MOF for effectively organic pollutants degradation and E. coli inactivation via catalytic activation of peroxymonosulfate," *Journal of the Taiwan Institute of Chemical Engineers* 132 (2022): 104154. http://dx.doi.org/10.1016/j.jtice.2021.11.021.

10. Ahmad, N., Khan, A. R., Siddiqui, M. R., Khan, M. D., Benamor, A., Anwer, A. H., Ahmad, N., "Advanced porphyrin-integrated Cu-MOF/semiconductor hybrids for high-efficiency removal of micropollutant via photocatalytic and adsorptive pathways," *Journal of Water Process Engineering*, 77, no (2025): 108447. http://dx.doi.org/10.1016/j.jwpe.2025.108447.

11. Wu, Z., Hou, L., Li, W., et al., "Application of a novel biomimetic double-ligand zirconium-based metal organic framework in environmental restoration and energy conversion," *Journal of Colloid and Interface Science* 610 (2022): 136-151. http://dx.doi.org/10.1016/j.jcis.2021.12.036.

12. Gan, X., Li, X., Wang, B., "Adsorption and degradation of multiple micropollutants with trace concentrations by porous Zr-porphyrin metal-organic frameworks with missing linker defects in water," *Microporous and Mesoporous Materials* 349 (2023): 112444. http://dx.doi.org/10.1016/j.micromeso.2023.112444.

13. Mi, X., Li, X., Wang, S., et al., "Efficient accumulation integrated with selective degradation of micropollutants under irradiation of visible light by the size-exclusion effect of metal–organic framework," *Separation and Purification Technology* 322 (2023): 124282. http://dx.doi.org/10.1016/j.seppur.2023.124282.

14. Xia, Z., Shi, B., Zhu, W., Lü, C., "Temperature-responsive polymer-tethered Zr-porphyrin MOFs encapsulated carbon dot nanohybrids with boosted visible-light photodegradation for organic contaminants in water," *Chemical Engineering Journal*, 426, no (2021): 131794. http://dx.doi.org/10.1016/j.cej.2021.131794.

15.	Li, W.-Q., Wang, Y.-X., Chen, J.-Q., Hou, N.-N., Li, Y.-M., Liu, X.-C., Ding, R.-R., Zhou, G.-N., Li, Q., Zhou, X.-G., Mu, Y., "Boosting photo-Fenton process enabled by ligand-to-cluster charge transfer excitations in iron-based metal organic framework," *Applied Catalysis B: Environmental*, 302, no (2022): 120882. http://dx.doi.org/10.1016/j.apcatb.2021.120882.

16. Gao, Y., Lu, J., Xia, J., Yu, G., "In Situ Synthesis of Defect-Engineered MOFs as a Photoregenerable Catalytic Adsorbent: Understanding the Effect of LML, Adsorption Behavior, and Photoreaction Process," *ACS Applied Materials & Interfaces*, 12, no (2020): 12706. http://dx.doi.org/10.1021/acsami.9b21122.

17. Wang, Z., Li, Q., Su, R., Lv, G., Wang, Z., Gao, B., Zhou, W., "Enhanced degradation of bisphenol F in a porphyrin-MOF based visible-light system under high salinity conditions," *Chemical Engineering Journal*, 428, no (2022): 132106. http://dx.doi.org/10.1016/j.cej.2021.132106.
